# Molecular Characterization of a Novel Family of *Trypanosoma cruzi* Surface Membrane Proteins (TcSMP) Involved in Mammalian Host Cell Invasion

**DOI:** 10.1371/journal.pntd.0004216

**Published:** 2015-11-13

**Authors:** Nadini Oliveira Martins, Renata Torres de Souza, Esteban Mauricio Cordero, Danielle Cortez Maldonado, Cristian Cortez, Marjorie Mendes Marini, Eden Ramalho Ferreira, Ethel Bayer-Santos, Igor Correia de Almeida, Nobuko Yoshida, José Franco da Silveira

**Affiliations:** 1 Departamento de Microbiologia, Imunologia e Parasitologia, Escola Paulista de Medicina, UNIFESP, São Paulo, Brasil; 2 The Border Biomedical Research Center, Department of Biological Sciences, University of Texas at El Paso, El Paso, Texas, United States of America; Harvard School of Public Health, UNITED STATES

## Abstract

**Background:**

The surface coat of *Trypanosoma cruzi* is predominantly composed of glycosylphosphatidylinositol-anchored proteins, which have been extensively characterized. However, very little is known about less abundant surface proteins and their role in host-parasite interactions.

**Methodology/ Principal Findings:**

Here, we described a novel family of *T*. *cruzi* surface membrane proteins (TcSMP), which are conserved among different *T*. *cruzi* lineages and have orthologs in other *Trypanosoma* species. TcSMP genes are densely clustered within the genome, suggesting that they could have originated by tandem gene duplication. Several lines of evidence indicate that TcSMP is a membrane-spanning protein located at the cellular surface and is released into the extracellular milieu. TcSMP exhibited the key elements typical of surface proteins (N-terminal signal peptide or signal anchor) and a C-terminal hydrophobic sequence predicted to be a trans-membrane domain. Immunofluorescence of live parasites showed that anti-TcSMP antibodies clearly labeled the surface of all *T*. *cruzi* developmental forms. TcSMP peptides previously found in a membrane-enriched fraction were identified by proteomic analysis in membrane vesicles as well as in soluble forms in the *T*. *cruzi* secretome. TcSMP proteins were also located intracellularly likely associated with membrane-bound structures. We demonstrated that TcSMP proteins were capable of inhibiting metacyclic trypomastigote entry into host cells. TcSMP bound to mammalian cells and triggered Ca^2+^ signaling and lysosome exocytosis, events that are required for parasitophorous vacuole biogenesis. The effects of TcSMP were of lower magnitude compared to gp82, the major adhesion protein of metacyclic trypomastigotes, suggesting that TcSMP may play an auxiliary role in host cell invasion.

**Conclusion/Significance:**

We hypothesized that the productive interaction of *T*. *cruzi* with host cells that effectively results in internalization may depend on diverse adhesion molecules. In the metacyclic forms, the signaling induced by TcSMP may be additive to that triggered by the major surface molecule gp82, further increasing the host cell responses required for infection.

## Author Summary

*Trypanosoma cruzi* is the etiologic agent of Chagas’ disease, which infects 6–7 million people worldwide, mostly in Latin America. Currently, there are no vaccines available, and the drugs used for treatment are toxic and are not fully effective. To infect mammalian hosts, *T*. *cruzi* relies on the ability to invade host cells, replicate intracellularly and spread the infection in different organs of the mammalian host. Knowledge of the structure and function of *T*. *cruz*i surface molecules is fundamental to understanding the mechanisms by which the parasite interacts with its host. *T*. *cruzi* infective forms engage a repertoire of surface and secreted molecules, some of which are involved in triggering signaling pathways both in the parasite and the host cell, leading to intracellular Ca^2+^ mobilization, a process essential for parasite internalization. Here, we described a novel family of *T*. *cruzi* surface membrane proteins (TcSMP), including their genomic distribution, expression and cellular localization. We studied the mechanism of action of TcSMP in host-cell invasion and proposed a triggering role for TcSMP in host-cell lysosome exocytosis during metacyclic internalization. TcSMP genes are conserved among different *T*. *cruzi* lineages and share orthologs in other *Trypanosoma* species. These results suggest that the diversification of TcSMP genes in mammalian trypanosomes occurred after continental drift. In *T*. *cruzi* this gene family expanded by gene duplication.

## Introduction

The kinetoplastid protozoan *Trypanosoma cruzi is* the etiologic agent of Chagas’ disease or American Trypanosomiasis which infects 6–7 million people worldwide, mostly in Latin America [1]. In the last few decades, the disease has spread to non-endemic regions, such as the United States and Europe, posing a new worldwide challenge [2]. *T*. *cruzi* is transmitted to humans by hematophagous triatomine vectors, that during blood meals, deposit feces containing the infective parasite forms, which enter the host through a lesion in the skin or mucosal surfaces. Other means of contamination include blood transfusion, congenital transmission and oral infection. In recent years, there have been frequent outbreaks of acute Chagas [3–8].

Host cell invasion is a crucial step for the establishment of *T*. *cruzi* infection. The first step of invasion is the adhesion of trypomastigotes to host cells by different surface molecules such as glycoproteins and proteases [9]. Among the most abundant surface molecules are glycoproteins anchored by the glycosylphosphatidylinositol (GPI) moiety and the GPI-related complex glycoinositolphospholipids [10, 11]. Abundantly expressed GPI-anchored surface glycoproteins, encoded by multigene families, include mucins, mucin associated surface proteins (MASP) and trans-sialidases (TS) [12–14]. These genes comprise approximately 17% of protein-coding genes in the *T*. *cruzi* genome and are involved in host-parasite interactions [12, 14–16]. Genomic comparison of the human parasites *T*. *cruzi*, *Trypanosoma brucei* and *Leishmania spp* revealed that most genes are common and arranged into syntenic regions [15, 17, 18]. However, genes encoding surface proteins are highly divergent, which is compatible with specific adaptations and survival strategies. Among these parasites, most species-specific genes encode surface antigens, such as variable surface glycoproteins (VSG) in *T*. *brucei*, TS and mucins in *T*. *cruzi* and promastigote surface proteases in *Leishmania spp* [15, 17].

While the major components of *T*. *cruzi* surface coat are well characterized, little is known about less abundant surface proteins and their roles in infection and transmission. Recently, low abundance surface proteins (SAP, TcTASV and DGF-1) have been characterized [19–24]. Screening of *T*. *brucei* cDNA libraries allowed the identification of a minor surface component of procyclic forms named Procyclic Surface Specific Antigen-2 (PSSA-2) [25]. PSSA-2 cDNA was isolated and predicted to encode a membrane-spanning protein with a C-terminal domain containing proline-rich tandem repeats. Fragoso *et al*. [26] demonstrated the importance of this cytoplasmic tail in targeting PSSA-2 to the plasma membrane and suggested that PSSA-2 could function as a sensor to transmit signals from the tsetse fly to the parasite. The PSSA-2 null mutant was fully competent to establishing midgut infections in tsetse, but was defective in colonizing the salivary glands and in producing infectious metacyclic forms [26]. In a previous study, we identified PSSA-2 orthologous proteins in *T*. *cruzi* epimastigotes and metacyclic trypomastigotes by tandem proteomic analysis while analyzing membrane enriched fractions [27]. In our study, we focused on characterizing PSSA-2 orthologous genes in *T*. *cruzi*, as well as studying their expression and potential functions in host parasite interactions. In the course of this study, we decided to name this new protein family the *T*. *cruzi* Surface Membrane Protein (TcSMP).

## Methods

### Ethics statement

All experiments involving animals were carried out under Brazilian National Committee on Ethics Research (CONEP) ethics guidelines, which are in accordance with international standards (CIOMS/OMS, 1985). The protocol was approved by the Ethical Committee of the Universidade Federal de São Paulo for animal experimentation (permit number: CEP 1877/08).

### Parasites, mammalian cell cultures and invasion assays

*Trypanosoma* species used in this study were: *T*. *cruzi* [clones CL Brener (CLB) and Dm28c, and CL strain], *T*. *cruzi marinkellei* and *T*. *brucei rhodesiense*. *T*. *cruzi* epimastigotes were maintained in axenic cultures at 28°C in liver-infusion tryptose (LIT) medium [28], supplemented with 10% heat-inactivated fetal calf serum (Vitrocell, Brazil). For *in vitro* metacyclogenesis, epimastigotes of the CL strain were harvested at early stationary phase (5–7 days) and allowed to differentiate in TC 100 medium (Vitrocell, Brazil). Metacyclic trypomastigotes were purified by chromatography on a DEAE-cellulose column (Sigma-Aldrich, St Louis, MO), as previously described [29]. Tissue culture trypomastigote (TCT) was obtained by differentiation of intracellular amastigotes in Vero cell monolayers infected with metacyclic trypomastigotes. Extracellular amastigotes were obtained by TCT differentiation in LIT medium as previously described [30]. Epimastigotes from *T*. *cruzi marinkellei* were maintained in LIT medium supplemented with 15% FCS at 28°C. Procyclic forms of *T*. *brucei rhodesiense* YTAT 1.1 were cultured in semi-defined medium (SDM-79) supplemented with 10% heat-inactivated fetal bovine serum (FBS) at 27°C.

Vero cells and human epithelial HeLa cells (Instituto Adolfo Lutz, São Paulo, Brazil) were grown at 37°C in a 5% CO_2_ humidified atmosphere in DMEM medium (Sigma-Aldrich, St Louis, MO) supplemented with 10% fetal bovine serum, 10 μg/mL streptomycin (Sigma-Aldrich, St Louis, MO), 100 U/mL penicillin (Sigma-Aldrich, St Louis, MO) and 40 μg/mL gentamicin (Sigma-Aldrich, St Louis, MO). Cell invasion assays were performed by seeding CL strain metacyclic forms onto 24-well plates containing 13-mm diameter round glass coverslips coated with 1.5 x 10^5^ HeLa cells. The multiplicity of infection (MOI) was 10. After 1 h of incubation with parasites, the duplicate coverslips were washed in PBS, fixed in Bouin solution, stained with Giemsa, and sequentially dehydrated in acetone, a graded series of acetone:xylol and xylol. The number of intracellular parasites was counted in 250 cells.

### Assay of TcSMP binding to host cell

Binding of the recombinant proteins TcSMP or GST to target cells was determined by ELISA. HeLa cells, grown in 96-well microtiter plates, were fixed with 4% paraformaldehyde in PBS for 30 min, washed and blocked with PBS containing 2 mg/ml BSA (PBS/BSA). Following 1 h incubation with the recombinant protein in PBS/BSA, cells were incubated sequentially with anti-TcSMP or anti-GST antibodies, and peroxidase-conjugated anti-rabbit IgG, all diluted in PBS/BSA. The final reaction was revealed by *o*-phenylenediamine, and the absorbance was read at 490 nm in ELx800TM absorbance microplate reader (BioTek, Winooski, VT).

### Cytoplasmic Ca^2+^ measurements by fluorescence microscopy

HeLa cells were grown overnight in DMEM with 10% fetal bovine serum on ibidi multichamber dishes (Hi-Q4, ibidi) and incubated at 37°C for 30 min with Fluo-4 Direct 2x (Invitrogen) diluted 1:1 in HBSS solution (Hank’s Balanced Salt Saline). Fluo-4 is a fluorescence indicator that binds intracellular free calcium ions. It presents a non-fluorescent AM grouping (acetoxymethyl ester) in its structure that when internalized, is cleaved by intracellular esterases and released to bind to cytoplasmic calcium. The emitted fluorescence intensity (500–550 nm) is related to the concentration of cytoplasmic calcium [31]. Images were captured with a microscope (Leica, Wetzlar, Germany) equipped with a HCX PL APO 40X dry objective 0.85 numerical aperture. The Fluo-4 probe was excited at l_Ex_ = 485/15 nm and light emission was detected at l_Em_ = 535/48 nm. Images were acquired at 2 sec intervals for 8 min (16 bit). The first two minutes corresponded to the basal fluorescence before application of the stimulus (recombinant TcSMP or GST). Fluorescence intensity was analyzed and normalized with reference to the basal fluorescence using Leica LAS AF software (Leica, Wetzlar, Germany).

### Genomic DNA isolation, Southern blot analysis and pulsed-field gel electrophoresis (PFGE)

Genomic DNA was isolated from 1 x 10^8^ epimastigotes of the clone CL Brener by homogenization in TELT buffer (50 mM Tris, pH 8.0; 62.5 mM EDTA, pH 9.0; 2.5 M LiCl; 4% Triton X-100; 20 μg/mL RNase), followed by extraction with phenol/chloroform and chloroform and precipitation with absolute ethanol. The pellet was washed with 70% ethanol, air-dried and suspended in TE (1 mM EDTA, pH 8.0; 10 mM Tris-HCl, pH 8.0).

Fifteen micrograms of genomic DNA were digested with 10 U of BamHI, BglII, EcoRI, EcoRV, HaeIII, HindIII, KpnI, PstI, SmaI or XhoI restriction enzyme (Invitrogen), and the fragments were resolved on a 0.8% agarose gel, stained with ethidium bromide (0.5 μg/mL) and photographed under UV light. Gels were sequentially treated with depurination solution (0.25 M HCl) for 45 min, denaturation solution (0.5 M NaOH; 1 M NaCl) for 20 min and neutralization solution (1 M Tris-base; 0.5 M NaCl) for 20 min and the DNA transferred to nylon membranes in 20x SSC for 2h. Membranes were prehybridized in the hybridization solution (50% formamide, 5x SSC, 5x Denhardt’s solution [Invitrogen], 0.1 mg/mL salmon sperm DNA, 0.5% SDS, 5 mM EDTA) for 2 h at 42°C and incubated in the hybridization solution containing ^32^P -labeled TcSMP derived probe for 16 h at 42°C. Membranes were washed twice in solution containing 2 x SSC, 0.1% SDS and 0.1% sodium pyrophosphate for 30 min at 42°C followed by two additional 30 min washes in 0.1 x SSC, 0.1% SDS and 0.1% sodium pyrophosphate at 56°C. Finally, membranes were exposed to X-ray film in light-tight cassettes at -70°C. The TcSMP probe (clone 23C) shares 99% identity with other TcSMP sequences deposited on TriTrypDB.

*T*. *cruzi* chromosomal DNA was resolved by pulsed-field gel electrophoresis (PFGE) in a Gene Navigator System (Pharmacia, Amersham, GE Healthcare Life Sciences) using a hexagonal electrode array as previously reported [32]. Plugs of agarose-embedded chromosomal DNA were prepared from epimastigotes of the clone CL Brener as previously described [32]. After electrophoresis, DNA was stained with ethidium bromide (0.5 μg/mL), transferred to nylon filters and hybridized as described above.

### Cloning and sequencing of TcSMP genes

To clone TcSMP genes from *T*. *cruzi* clone CL Brener, four oligonucleotide primers (P1F, P2F, P3R and P4R) were designed based on conserved regions of sequences deposited in the TriTrypDB (S1 Fig). PCR amplification was carried out with genomic DNA as follows: initial denaturation (5 min at 94°C), 40 cycles of denaturation (30 s at 94°C), annealing (1 min at melting temperature) and elongation (30 s at 72°C). PCR products were resolved in 0.8% agarose gels and purified using the Wizard SV Gel and PCR Clean-up System (Promega, Madison, WI). Purified amplicons were cloned into the pGEM-T Easy vector (Promega, Madison, WI), transformed into *Escherichia coli* DH5α strain and sequenced by the dye terminator method on an ABI PRISM 3130xl Genetic Analyzer (Applied Biosystems, Foster City, CA).

### Sequence alignment, syntenic and phylogenetic analyses

Similarity searches using BLASTN and BLASTP [33] algorithms were carried out to search for TcSMP nucleic acid and protein sequences of *T*. *cruzi* in the TriTrypDB genomic resource (http://www.tritrypdb.org) and GenBank. Alignment was performed using sequences from clone CL Brener excluding the truncated sequences. Nucleotide and deduced amino acid sequences were aligned using ClustalW [34] and manually adjusted. The search for orthologous genes in other trypanosomatids was performed using BLASTn and BLASTp algorithms. The TcSMP sequences from *T*. *cruzi* (clones CLB, Sylvio X/10 and PCR amplified products from CL Brener), *T*. *cruzi marinkellei*, *T*. *brucei* (strains Lister 427 and TREU927), *T*. *brucei gambiense*, *T*. *congolense* and *T*. *vivax* were aligned using the MUSCLE algorithm [35] and manually inspected with Seaview (4.0 version) [36]. Truncated sequences were excluded from ML analysis. The TcSMP phylogenetic analysis was inferred with maximum likelihood (NJ) method, using the LG substitution model with a bootstrap value of 1,000 replicates.

Gene synteny analysis was carried out between chromosomes containing TcSMP orthologous genes from different trypanosomatids. Regions were aligned in pairs in the BLASTN program and analyzed by the ACT program. Analyses were conducted via TBlastN using the Artemis Comparison Tool (ACT) [37].

The prediction of functional domains and posttranslational modifications was performed using bioinformatics tools available at the ExPASy proteomic server (http://www.expasy.org). Signal peptide and signal anchor were determined using SignalP3.0 and SignalP4.0. SignalP3.0 also reports the probability of signal anchor, which is also named uncleaved signal peptide [38]. Transmembrane helices, *O*-, *N*-glycosylation and phosphorylation sites were determined using TMHMM, NetOGlyc, NetNGlyc and NetPhos software, respectively.

### Expression and purification of recombinant TcSMP

A 311-bp EcoRI fragment from clone 23B (see S5 Fig) was inserted into pGEX-1λT to produce the TcSMP-GST fusion protein. *E*. *coli* BL21 bacteria transformed with this construct were grown in LB medium, induced with 1 mM isopropyl β-D-1-thiogalactopyranoside (IPTG) for 3 h at 37°C and harvested by centrifugation. Cells were suspended in PBS containing protease inhibitors and sonicated for 10 min on ice. Extracts were centrifuged at 1,600 x g for 20 min at 4°C to separate the insoluble proteins (pellet) from the soluble proteins (supernatant). The pellet was suspended in Laemmli’s sample buffer and resolved on 10% SDS-PAGE. Gels were stained with ice-cold 250 mM KCl and the bands corresponding to the recombinant protein were excised from the gels. Gels slices were dialyzed against ammonium bicarbonate for 48 h at 4°C under agitation followed by 36 h at 4°C against distilled water. Purification was checked by SDS-PAGE stained with colloidal Coomassie Blue and immunoblotting. Purified protein was quantified with Coomassie Plus (Pierce, Thermo Fisher Scientific) in 96-well plates at 620 nm.

### Antibody production

Anti-TcSMP polyclonal antibodies were obtained by immunization of 6 weeks old BALB/c mice with four doses of purified TcSMP recombinant protein via the intraperitoneal route. Each mouse received a first dose consisting of 50 μg of antigen with aluminum hydroxide as adjuvant, followed by another three doses of 25 μg each, under the same conditions. The doses were given at 15 days intervals and the mice were bled two weeks after the fourth dose. Rabbits were immunized intradermally with 1 mg of TcSMP recombinant protein (protein concentration 1 mg/mL) emulsified with Freund’s adjuvant (Sigma-Aldrich, St Louis, MO), followed by another three doses of 1 mg each, under the same conditions. The doses were given at 15 days intervals and the rabbits were bled two weeks after the fourth dose. Sera were separated by centrifugation and kept frozen at -20°C.

In order to verify the specificity of the antibodies used in this study, we carried out careful control experiments to ensure that they recognized the correct protein. First of all, the polyclonal antibodies were purified after incubation with the purified recombinant TcSMP in phase with GST immobilized on nitrocellulose membrane followed by elution with glycine. Next, we performed western assays by incubating the immunopurified antibodies against TcSMP-GST and MVK (mevalonate kinase) with recombinant proteins (S2A Fig). Anti-TcSMP specifically reacted against TcSMP while anti-MVK recognized only the MVK protein.

As an additional control, the coding DNA sequence of the TcSMP (TcCLB.510129.30) gene was synthetized and cloned in phase with GFP into pTREX-GFP vector [39]. Epimastigotes (CL strain) were electroporated with 70 μg of the construction TcSMP-GFP by two pulses of 0.3 kV/ 500 μF in the BioRad Gene Pulser apparatus. Transfected cells were selected in the presence of 500 μg/mL G418 in LIT medium supplemented with 20% heat-inactivated fetal calf serum (Vitrocell, Brazil). Parasites expressing TcSMP-GFP were incubated with anti-TcSMP antibodies following the same protocol described in the item “immunofluorescence” (methods section). Confocal images obtained from each fluorescence channel were overlapped, and co-localized pixels are shown in panel CP (S2B Fig). The presence of co-localized pixels between fluorescence emission of GFP (TcSMP-GFP) and Alexa Fluor 488-labeled anti-mouse immunoglobulins confirmed the antibody specificity for TcSMP protein. Our results with transfected parasites corroborated the immunofluorescence staining pattern obtained with anti-TcSMP antibodies as the recombinant protein TcSMP-GFP displayed the same labeling pattern.

### Western blot analysis

*T*. *cruzi* developmental forms (1 x 10^7^ cells) and procyclic forms of *T*. *brucei* (1x10^7^ cells) were washed in PBS and incubated in 4 x Laemmli sample buffer for 5 min at 100°C. Proteins were separated in 10% SDS-PAGE, transferred to Hybond ECL membranes (Amersham, GE Healthcare Life Sciences) and blocked with PBS/7% skim milk overnight at 4°C. Membranes were incubated with polyclonal anti-TcSMP antibody (1:200) for 3 h, washed with PBS/0.005% Tween 20 and incubated for 1 h with a secondary goat anti-mouse antibody (H+L) (1:10,000) (Bio-Rad). The immunocomplexes were detected by chemiluminescence using the ECL-Plus Western Blot Detection System (Amersham, GE Healthcare Life Sciences).

### Immunofluorescence and flow cytometry

*T*. *cruzi* developmental forms were harvested from culture medium and washed with PBS. Live parasites were incubated in 1% BSA for 10 min on ice followed by 1 h with anti-TcSMP antibodies diluted 1:20 in 1% BSA, washed with PBS and fixed in 4% formaldehyde for 15 min on ice. Thereafter, parasites were incubated with fluorescein-labeled goat anti-mouse IgG (Sigma-Aldrich, St Louis, MO) diluted 1:100 in BSA and 1 mM DAPI (4',6-diamidino-2-phenylindole; Molecular Probes). After three washes with PBS, the coverslips were mounted in glycerol buffered with 0.1 M Tris pH 8.6 containing 0.1% *p*-phenylenediamine. Confocal images were obtained using a Bio-Rad 1024UV system coupled to a Zeiss Axiovert 100 microscope or a Leica TCS SP5 II system. Images were acquired with 100X (1.4 NA) oil immersion objectives. In parallel, parasites were first fixed with 4% formaldehyde for 15 min at room temperature and then processed as described above, except by the fact that parasites were incubated by a 1 h with anti-TcSMP followed by 1 h incubation with fluorescein-labeled goat anti-mouse IgG antibody in the presence of 0.1% saponin (0.15% gelatin in PBS containing 0.1% sodium azide and 0.1% saponin).

Flow cytometry experiments were performed following the procedures described above. Parasites incubated without antibody or only with the fluorescein-labeled goat anti-mouse IgG antibody were included as controls for auto-fluorescence and background, respectively. The number of fluorescent parasites was estimated with a FACScanto cytometer (BD Biosciences, Franklin Lakes, NJ).

In parallel, fixed parasites were placed in 12-well slides for immunofluorescence reaction (IF reaction). The wells were washed with PBS and blocked in PBS/1% BSA solution for 30 min. Parasites were incubated with anti-TcSMP (1:20) antibodies, washed with PBS and incubated for 1 h with an Alexa Fluor 568 anti-mouse IgG antibody made in goat solution diluted 1:100 in 0.1% saponin and 1 mM DAPI (4'6 -diamino-2-phenylindole, Molecular Probes). Finally, the wells were washed with PBS and the slides were mounted using Glycerol-PPD. The fluorescence was observed by confocal microscopy using the Bio-Rad 1024UV system adapted for a Zeiss Axiovert 100 microscope with Leica TCS SP5 system or II. Images were acquired with a 63X objective with immersion oil.

## Results

### Identification and classification of the TcSMP gene family

PSSA-2 orthologs identified in *T*. *cruzi* genomes have been annotated as “procyclic form surface glycoprotein” or “procyclic form surface phosphoprotein” in the TriTryp and GenBank databases. In the present work we demonstrate that these proteins are associated with cellular membranes and are expressed at the cell surface of all *T*. *cruzi* developmental forms. For this reason, it is more appropriate to refer to these proteins collectively as *T*. *cruzi* Surface Membrane Proteins (TcSMP). With the aim of characterizing the TcSMP gene family, we used the BLASTP algorithm to retrieve sequences from the TriTrypDB and GenBank databases by entering the surface glycoprotein (XP_803885.1) and procyclic form surface phosphoprotein (XP_819855.1) of *T*. *cruzi* clone CL Brener (CLB) as queries. We identified 19 TcSMP genes in the *T*. *cruzi* genome database whose products share approximately 40% identity with the *T*. *brucei* PSSA-2 protein, distributed as follows: 9 sequences in CLB, 3 in clone Sylvio X10/4, 2 in clone Dm28c and 5 in *T*. *cruzi marinkellei* (Table 1).

**Table 1:** Identification and classification of Tc-SMP genes from *T*. *cruzi* genomes deposited in TriTrypDB and GenBank.

Locus ID ^1^	Annotation	CDS ^2^ (size bp)	Molecular Mass ^3^ (kDa)	Tc-SMP Group ^4^	*T*. *cruzi* isolate
		
TcCLB.509639.10	Procyclic form surface glycoprotein	1,413	51.3	Tc-SMP_L	CL Brener
TcCLB.510129.20	Surface glycoprotein	1,380	49.9	Tc-SMP_L	CL Brener
TcCLB.510129.30	Surface glycoprotein	1,380	49.9	Tc-SMP_L	CL Brener
TcCLB.507711.100	Procyclic form surface glycoprotein	1,326	47.9	Tc-SMP_L	CL Brener
TcCLB.507711.110	Procyclic form surface glycoprotein	1,263	45.7	Tc-SMP_L	CL Brener
TcCLB.507711.90	Procyclic form surface glycoprotein	1,206	43.4	Tc-SMP_S	CL Brener
TcCLB.508173.120	Trans-sialidase	1,143	41.8	Tc-SMP_S	CL Brener
KJ682657 ^5^	Surface Membrane Protein (clone 23B)	1,160	41.4	ND ^6^	CL Brener
KJ682658 ^5^	Surface Membrane Protein (clone 24B)	1,050	37.9	ND ^6^	CL Brener
KJ682659 ^5^	Surface Membrane Protein (clone 24D)	1,052	37.9	ND ^6^	CL Brener
TcCLB.510129.11	Surface glycoprotein	557	18.2	Truncated ^7^	CL Brener
TcCLB.509637.36	Procyclic form surface glycoprotein	501	21.7	Truncated ^7^	CL Brener
TCSYLVIO_001920	Trans-sialidase	1,140	41.7	Tc-SMP_S	Sylvio X10/4
TCSYLVIO_009546	Surface glycoprotein	942	34	Truncated ^7^	Sylvio X10/4
TCSYLVIO_000501	Surface glycoprotein	645	23.3	Truncated ^7^	Sylvio X10/4
TCDM_04964	Surface glycoprotein	1,313	47.5	Tc-SMP_L	Dm28c
TCDM_01936	Trans-sialidase	662	24.1	Pseudogene	Dm28c
Tc_MARK_3605	Surface glycoprotein	1,314	47.8	Tc-SMP_L	*T*. *cruzi marinkellei*
Tc_MARK_706	Trans-sialidase	1,302	47.2	Tc-SMP_L	*T*.*cruzi marinkellei*
Tc_MARK_3606	Procyclic form surface glycoprotein	1,205	43.1	Tc-SMP_S	*T*. *cruzi marinkellei*
Tc_MARK_7644	Surface glycoprotein	750	27.2	Truncated ^6^	*T*. *cruzi marinkellei*
Tc_MARK_785	Surface glycoprotein	605	21.6	Truncated ^7^	*T*. *cruzi marinkellei*

^1^ TriTrypDB entries are prefixed by species name they were derived. TcCLB, *T*. *cruzi* clone CL Brener; TCSYLVIO, *T*. *cruzi* clone Sylvio X/10A; TCDM, *T*. *cruzi* clone Dm28c; TC_MARK, *T*. *cruzi* marinkellei.

^2^ Coding DNA Sequence.

^3^ Predicted molecular mass of Tc-SMP proteins by coding region translation.

^4^ Classification of Tc-SMP genes according to CDS size: Tc-SMP_L (large)–CDS = 1,263–1,380 bp and Tc-SMP_S (small)–CDS = 1,140–1,205 bp.

^5^ Tc-SMP sequences isolated in this work indicated by GenBank accession numbers.

^6^ Not determined.

^7^ Truncated protein due to incomplete assembly in the draft genome.

Gene identification in parasite genomes was performed by automatic annotation software that can produce some erroneous predictions. To rule out errors in prediction and annotation, TcSMP sequences were also manually curated. The initial predicted CDSs for the TcCLB.507711.100 and TcCLB.507711.110 genes missed a 44- and a 34-amino acid-extensions at N-terminus, respectively (S1 Fig). In this study we used the correct CDSs, which can be found in S3 Fig. In addition four sequences were clearly misannotated as TS (TcCLB.508173.120, TCSYLVIO_001920, TCDM_01936, Tc_MARK_706) (Table 1), besides their lack of similarity to the TS gene, they share 70–80% similarity with the TcSMP genes (Table 2).

**Table 2:** Tc-SMP proteins secreted into the extracellular medium by epimastigotes and metacyclic trypomastigotes (clone Dm28c) identified by proteomic analysis.

Peptide sequence	Accession number^(1)^	Sample	Xcorr^(2)^	DC*n* ^(3)^
YVTASVVYPPVGAKNLRVEVDIR	TvY486_1010870	Epi-V16^(4)^	2.57	0.179
GSLNVPGYSGVPTRYVTASVVYPPVGAK	TvY486_1010870	Epi-VF^(5)^	2.76	0.274
DCKSRLNCQCNELLNSFMNQCVASGGK	TcCLB.509637.36	Epi-VF^(5)^	3.13	0.274
	TcCLB.507711.90			
	TcCLB.510129.20			
	TCDM_04964			
PNKKSLGTPLYLAPASQGILTSGGPGDTAPNPYAHLAEQTR	TcCLB.508173.120	Meta-V16^(4)^	2.47	0.147

^(1)^ TriTrypDB entries are prefixed by the species name they were derived: TvY486_ for T. vivax; and TcCLB and TCDM_ for T. cruzi clone CL Brener and Dm28c, respectively.

^(2 and 3)^ To validate the quality of protein identification, the following parameters were used: Xcorr (CrossCorr / avg [AutoCorr offset = -75 to 75]) ≥ 1.5, 2.0, and 2.5, for singly, doubly and triply charged peptides, respectively. DC*n* (Xcorr_1_ –Xcorr_2_ / Xcorr1) ≥ 0.1.

^(4)^ Sample enriched in plasma membrane-derived vesicles or ectosomes (V16) from epimastigotes (Epi) or metacyclic trypomastigotes (Meta).

^(5)^ Sample enriched in soluble proteins (vesicle-free, VF) from epimastigotes (Epi).

TcSMP genes were classified into two groups based on the length of their coding sequences (CDS): TcSMP_L comprising a large CDS encoding 45.7 to 51.3 kDa proteins and TcSMP_S comprising a small CDS encoding 41.7 to 43.4 kDa proteins (Table 1). The predicted molecular masses of TcSMP proteins listed in Table 1 were based on the translation of each open reading frame without considering additional processing of the protein, i.e., removal of the signal peptide of TcSMP_L members after trafficking through the endoplasmic reticulum, as discussed below. Seven TcSMP truncated sequences were found in the *T*. *cruzi* genome, one was a pseudogene and the remaining sequences were truncated (Table 1), as judged by the existence of gaps in the genome assembly. Sequence analysis of TcSMP genes showed a high degree of conservation between CLB sequences, ranging from 82 to 98% identity (S1 Fig). PCR amplification using primers designed based on conserved regions of TcSMP genes allowed the identification of three new members of the TcSMP family in the CLB (GenBank KJ682657, KJ682658 and KJ682659), which exhibited 88–91% similarity with previously identified genes but also displayed nucleotide differences, which indicates that they correspond to new copies of TcSMP (S1 Fig).

### Identification of structural features of TcSMP proteins

**Fig 1:**
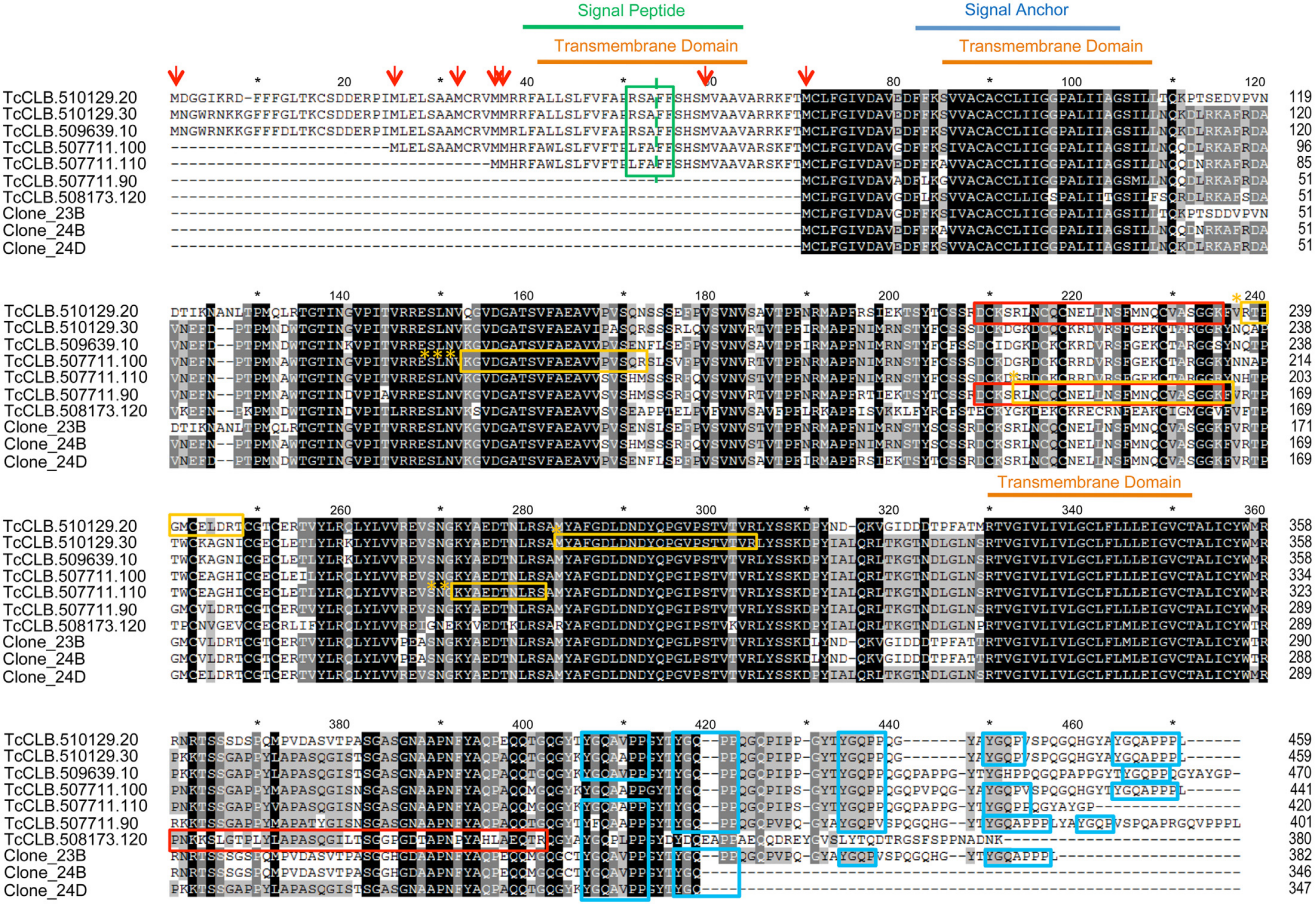
Alignment of TcSMP amino acid sequences of *T*. *cruzi—*clone CL Brener (CLB). Sequences TcCLB.510129.20, TcCLB.510129.30, TcCLB.509639.10, TcCLB.507711.90, extended TcCLB.507711.100, extended TcCLB.507711.110 and TcCLB.508173.120 were deposited in the GenBank database by the *T*. *cruzi* Genome Project. Clones 23B, 24B and 24C (GenBank KJ682657, KJ682658 and KJ682659) were isolated in this work from genomic DNA (CLB) by PCR. Sequences were aligned by the MegAlign program (DNASTAR). Black, gray and light gray blocks indicate residues with 100%, 80% and 60% identity, respectively. Colored lines above the sequences denote the predicted signal peptide in green, transmembrane domains in orange and uncleaved anchor signal in blue. Red arrows indicate the putative initiator methionine. Yellow boxes correspond to peptides found in the proteomic analysis of *T*. *cruzi* membrane protein-enriched fractions [27]. Red boxes correspond to peptides identified in the *T*. *cruzi* secretome [43]. Blue boxes correspond to the YGQ motif present in Procyclic Surface Specific Antigen-2 (PSSA-2) of *T*. *brucei*.

TcSMP genes shared 82–98% nucleotide identity (S1 Fig), whereas amino acid identity varied from 70–90% and 65–93% for TcSMP_L and TcSMP_S, respectively (Fig 1). Sequence alignment showed that TcSMP_L proteins share an N-terminal extension of 69 amino acids containing seven in frame putative initiator codons. The region immediately after the fourth ATG codon encodes a typical 25-amino acid signal peptide with a putative cleavage site between residues RSA-FF (Fig 1). Upstream sequences in the vicinity of the third ATG best fit the Kozak consensus sequence (S4 Fig). Among other aspects of the mRNA structure, the context surrounding the AUG codon can modulate the initiation of translation [40]. The Kozak sequence located upstream of the initiation codon is expected to facilitate ribosome binding and thus the beginning of protein synthesis. In view of this, we suggest that the translation of large sequences initiates at the fifth methionine. The presence of several initiation codons in the same reading frame is common among *T*. *cruzi* surface protein genes such as GP82 and GP90 [41, 42].

**Fig 2:**
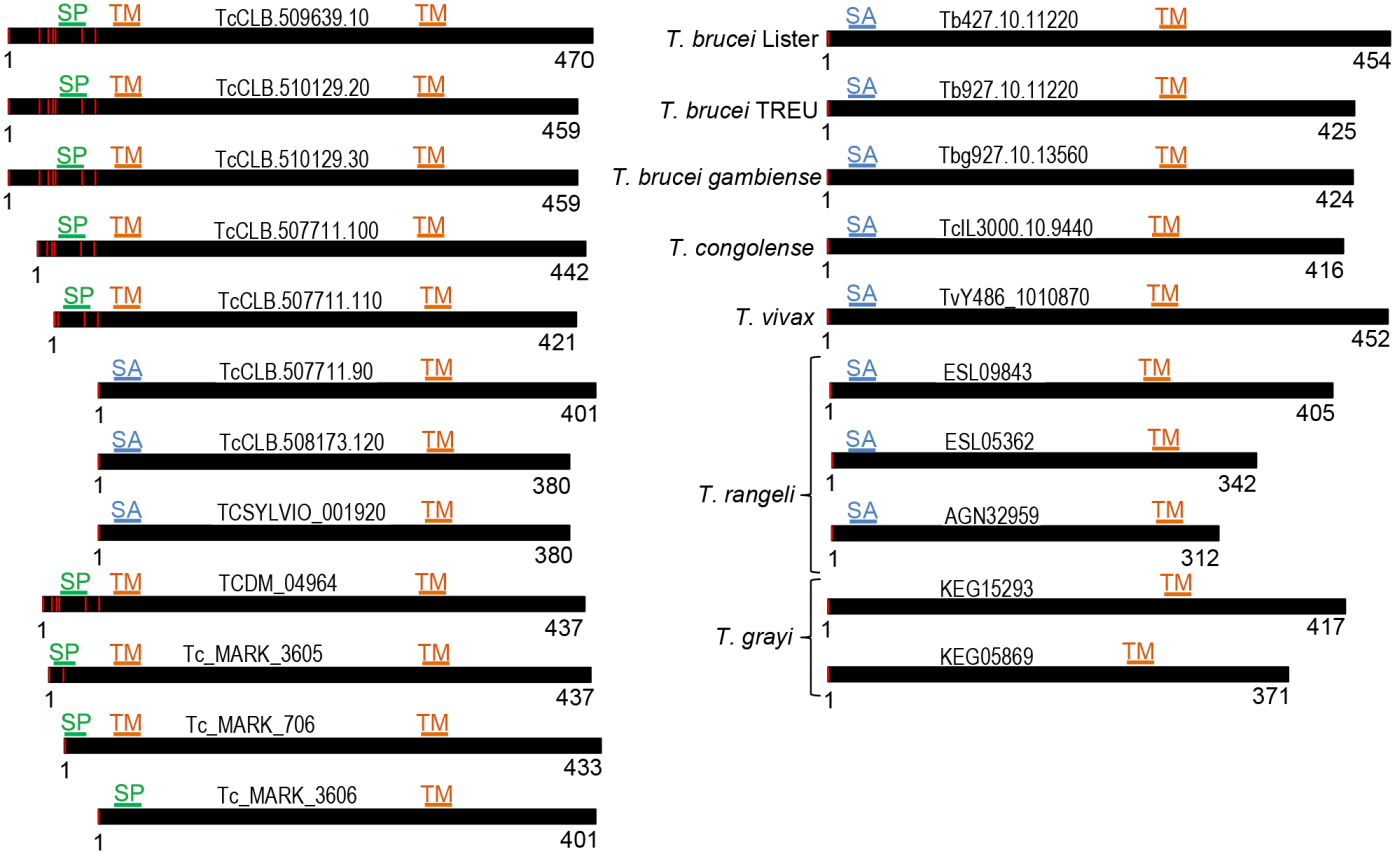
Modular architecture of TcSMP paralogs and orthologs. Left Panel) Schematic representation of TcSMP proteins from *T*. *cruzi* isolates (CLB, Sylvio X10/1 and Dm28c) and *T*. *cruzi marinkellei*. Right Panel) Schematic representation of TcSMP orthologs in *T*. *brucei*, *T*. *brucei gambiense*, *T*. *congolense*, *T*. *vivax*, *T*. *rangeli* and *T*. *grayi*. The amino acid sequence is represented by a black bar and the first and last residues are indicated below. The red vertical lines indicate the putative in frame initiator methionine residues. The positions of predicted signal peptide (SP), signal anchor (SA) and transmembrane domain (TM) are indicated by green, blue and orange lines, respectively. Sequences were identified by their entries in TriTrypDB, with the exception of those from *T*. *rangeli* and *T*. *grayi*, which were identified by GenBank accession numbers. *T*. *cruzi* sequences: TcCLB, clone CL Brener; TCSYLVIO, clone Sylvio X10/1; TCDM, clone Dm28c; Tc_MARK, *T*. *cruzi marinkellei*.

TcSMP proteins have 2–3 hydrophobic domains at the N- and C-termini (Fig 1 and S5 Fig). Considering that the translation of TcSMP_L starts at the 3^rd^ methionine, the first hydrophobic domain corresponds to a typical amino-terminal signal peptide with basic residues followed by a hydrophobic stretch with a putative cleavage site RSA-FF (Fig 1). TcSMP_S proteins have two transmembrane domains and the first one is located immediately after the initiator methionine, which was predicted to be a signal anchor [38]. An exception was Tc_MARK_3606 in which the first hydrophobic domain was predicted to be a signal peptide (Fig 2). A similar pattern is present in PSSA-2 of *T*. *brucei* (S5 Fig) which is attached to the plasma membrane by a stable transmembrane anchor characteristic of membrane proteins [25]. The C-terminus of TcSMP contains 4 proline residues centered on the YGQ motif that can also contribute to size differences among the TcSMP proteins (Fig 1). Interestingly, there are 8 tandem proline repeats on the cytoplasmic tail of PSSA-2 that are predicted to form tight helices [25]. TcSMP and PSSA-2 repeats share low degrees of similarity.

Systematic BLAST searches revealed the presence of TcSMP_S orthologs in the genomes of *Trypanosoma rangeli* and mammal-dwelling African trypanosomes. The identified TcSMP orthologs are depicted with selected examples from other species in Fig 2. *Trypanosoma grayi*, a trypanosome from crocodilians, has a single transmembrane domain at the C-terminus (Fig 2).

### Expression of TcSMP proteins

**Fig 3:**
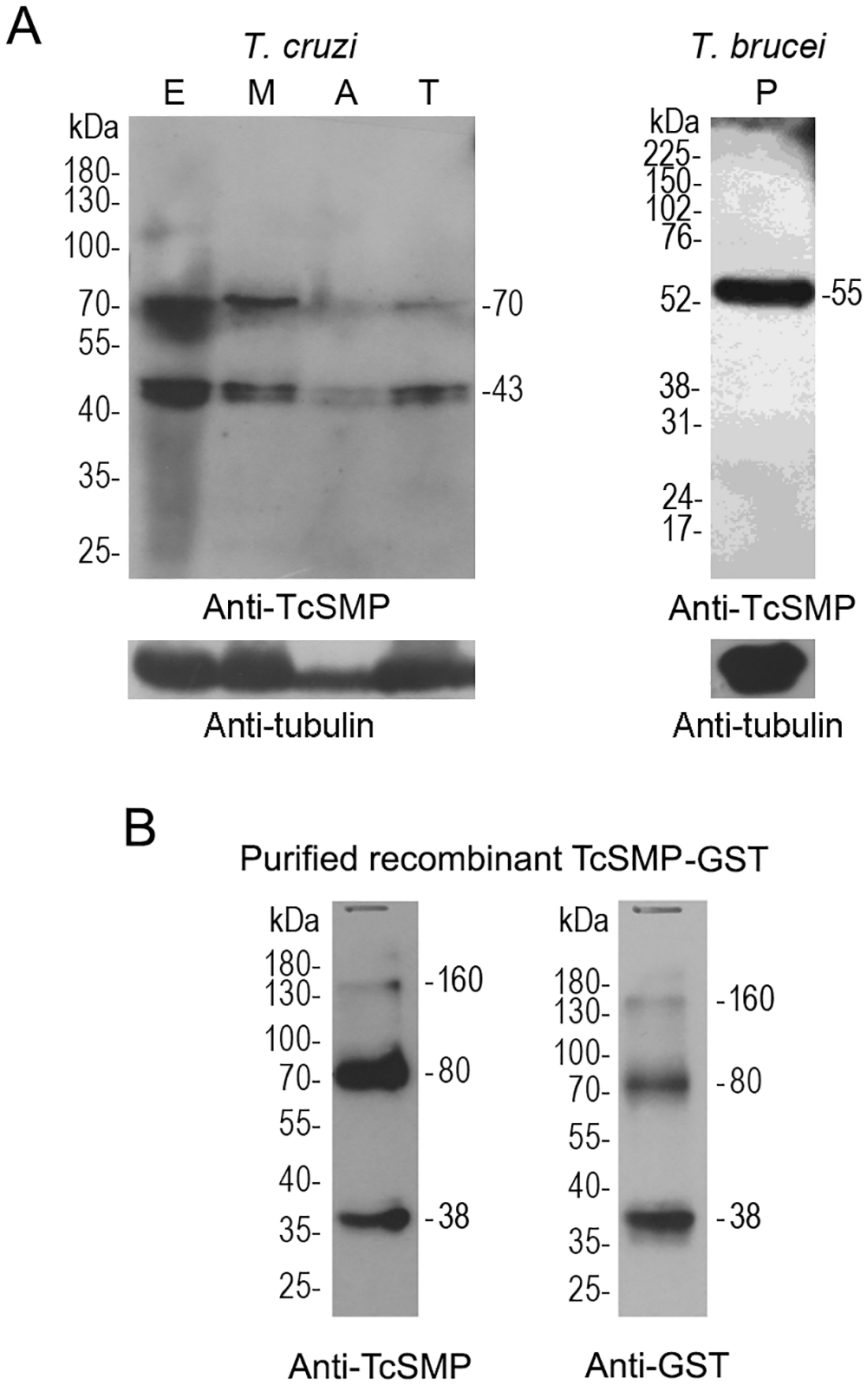
Western blotting analysis of TcSMP proteins in the different developmental stages of *T*. *cruzi* and in the procyclic form of *T*. *brucei rhodesiense*. A) Samples of *T*. *cruzi* (CL strain) (1 x 10^7^ cells/slot) and *T*. *brucei* (5 x 10^6^ cells/slot) were lysed in Laemmli sample buffer. Proteins were separated by electrophoresis on SDS- polyacrylamide gel (10%), transferred to a nitrocellulose membrane and reacted against anti-TcSMP polyclonal antibodies. Antibody against a constitutively expressed protein (tubulin) was used as a loading control. *T*. *cruzi* developmental forms: epimastigotes (E), metacyclic trypomastigotes (M), extracellular amastigotes (A) and tissue culture trypomastigotes (T). *T*. *brucei rhodesiense* procyclic forms. B) Two μg of the recombinant TcSMP-GST purified protein was separated by SDS-PAGE and reacted against anti-TcSMP and anti-GST antibodies. The recombinant protein is coded by a 311-bp fragment of TcSMP in phase with GST (see methods and S4 Fig). Molecular mass markers in kilodaltons (kDa) are indicated on the left and reactive proteins molecular mass on the right.

Expression of TcSMP was analyzed using polyclonal antibodies raised against a recombinant protein carrying a small fragment of TcSMP that encodes a predicted transmembrane helix flanked by cytoplasmic and extracellular hydrophilic regions (see S5 Fig). Western blot analysis of whole parasite extracts using mouse anti-TcSMP polyclonal antibodies detected a ~43 kDa protein band expressed in all parasite developmental forms, which is the predicted molecular mass for TcSMP_S (Fig 3). Assuming that TcSMP_L has a signal peptide that is removed after trafficking through the endoplasmic reticulum, all members of this family can be expected to generate proteins of the same molecular mass as TcSMP_S. An additional ~70 kDa band consistently reacted against anti-TcSMP antibody; this size is close to that of two of these proteins together. Fragoso et al. [26] found some evidence that PSSA-2 forms homodimers or multidimers. As with its ortholog, there are few cysteine residues in the TcSMP protein sequence that could be responsible for the formation of disulfide bridges. Although the sample buffer contains 2-mercaptoethanol or dithiothreitol (DTT), our protein samples were not prepared with antioxidants; hence, intermolecular disulfide bonds could be reconstituted during electrophoresis. Interestingly, when we performed a western blot containing the recombinant TcSMP protein excised from a single 38 kDa band of bacterial extract, two larger bands (~80 kDa and ~160 kDa) reacted against anti-TcSMP antibody after SDS-PAGE (Fig 3). It is possible that the larger bands are dimer/multidimers and that this could be the stable conformation of native TcSMP. Whilst some evidence supports the hypothesis that this band may be a dimer, further experiments should be carried out to prove this hypothesis.

The PSSA-2 coding region size can vary in *T*. *brucei* isolates mostly due to insertion/deletion of tyrosine/proline-rich repeats. The predicted size of these sequences in *T*. *brucei*_TREU927 and *T*. *brucei gambiense*_DAL972 was 47 kDa according to the translation of the open reading frame, while the predicted size for PSSA-2 in *T*. *brucei* Lister (strain 427) was 50 kDa, the same size reported by Fragoso et al., 2009 [26] who used *T*. *brucei* AnTat 1.1 in western assays. Anti-TcSMP antibodies recognized a 55 kDa protein in *T*. *brucei* procyclic forms due to the amino acid-conserved region between TcSMP and PSSA-2 used for immunization procedures. As the *T*. *brucei* rhodesiense genome has not yet been sequenced, we cannot predict the exact protein size. In addition, we do not have any information as to whether there are glycosylation sites or whether there were any other post-translational modifications in the processed protein that could justify its larger size compared with PSSA-2 AnTat 1.1.”

Recently, Bayer-Santos et al. [43] performed a proteomic analysis of the *T*. *cruzi* secretome in which two populations of extracellular vesicles (exosomes and plasma membrane-derived vesicles or ectosomes) and soluble proteins released by epimastigotes and metacyclic trypomastigotes were characterized. Although TcSMP proteins were not reported in this study [43], we decided to revisit our raw data to search for TcSMP proteins among those identified by only one peptide, which were excluded from the previous work in which only proteins identified with two peptides were considered. Four peptides were identified in samples from epimastigotes and metacyclic trypomastigotes, either in the soluble protein fraction or the small membrane vesicle fraction (fraction V16) (Fig 1 and Table 2). Two peptides identified in epimastigotes share identity to an ortholog protein from *T*. *vivax* (TvY486_1010870), which may indicate the existence of new TcSMP genes in *T*. *cruzi*. These data suggest that TcSMP proteins and their orthologs can be secreted by *T*. *cruzi* and *T*. *vivax* (Fig 1 and Table 2).

### Subcellular localization of TcSMP in different developmental stages of the *T*. *cruzi* life cycle

**Fig 4:**
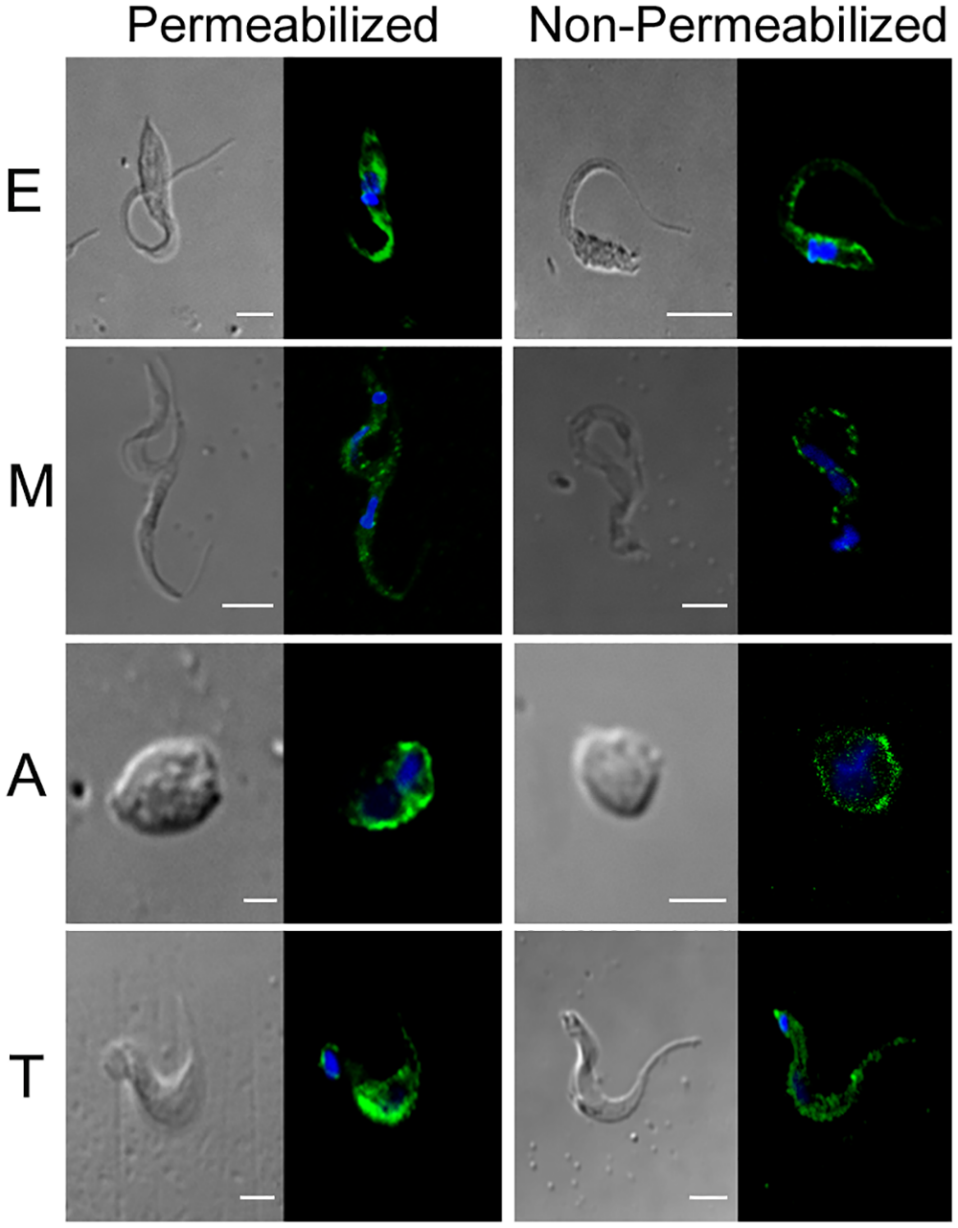
Cellular distribution of TcSMP proteins in different stages of the *T*. *cruzi* life cycle. Indirect immunofluorescence with anti-TcSMP antibodies in permeabilized (left) or non-permeabilized (right) *T*. *cruzi* (CL strain). *T*. *cruzi* developmental forms: epimastigotes (E), metacyclic trypomastigotes (M), extracellular amastigotes (A) and tissue culture trypomastigotes (T). Labeling with DAPI and TcSMP proteins is shown in blue and green, respectively. Bar: 10 μm.

Live and permeabilized parasites were analyzed by indirect immunofluorescence, using anti-TcSMP antibodies (Fig 4). TcSMP distribution varied from dispersed throughout the cytosol in permeabilized parasites to punctate and concentrated in discrete spots on the cell surface of live parasites. From these results, we conclude that TcSMP proteins are located on the surface as well as intracellularly in *T*. *cruzi* developmental forms. Surface proteins follow the parasite’s secretory pathway through the endoplasmic reticulum (ER), where the signal peptide is removed before being addressed to the cell membrane. To confirm that the signal peptide directs TcSMP to the ER, parasites expressing TcSMP-GFP were incubated with anti-BIP, an endoplasmic reticulum marker. Overlapping of confocal images obtained from each fluorescence channel showed co-localized pixels between TcSMP-GFP and anti-BIP (S2B Fig). This result suggests that TcSMP goes to the ER and then it is addressed to the cell surface membrane. The PSSA-2 protein was located on the surface of *T*. *brucei* procyclic forms of parasites transfected with the complete ORF protein [26]. We confirmed the presence of TcSMP and PSSA-2 on the cell surface of *T*. *cruzi* tissue culture trypomastigotes (TCT) and *T*. *brucei* procyclic forms by flow cytometry analysis, labeling live and permeabilized parasites with anti-TcSMP antibodies. S6 Fig shows the labeling of live *T*. *cruzi* TCT forms and *T*. *brucei* procyclic forms with anti-TcSMP. Approximately 90%-92% of live *T*. *cruzi* and *T*. *brucei* parasites labeled with anti-TcSMP antibodies exhibited greater fluorescence intensity than those incubated with pre-immune serum. Besides indicate that anti-TcSMP antibodies recognized an ortholog in the *T*. *brucei* surface membrane, these results confirm that TcSMP is located on the cell surface of *T*. *cruzi* tissue culture trypomastigotes (TCT).

### Involvement of TcSMP in the interaction with host cells

**Fig 5:**
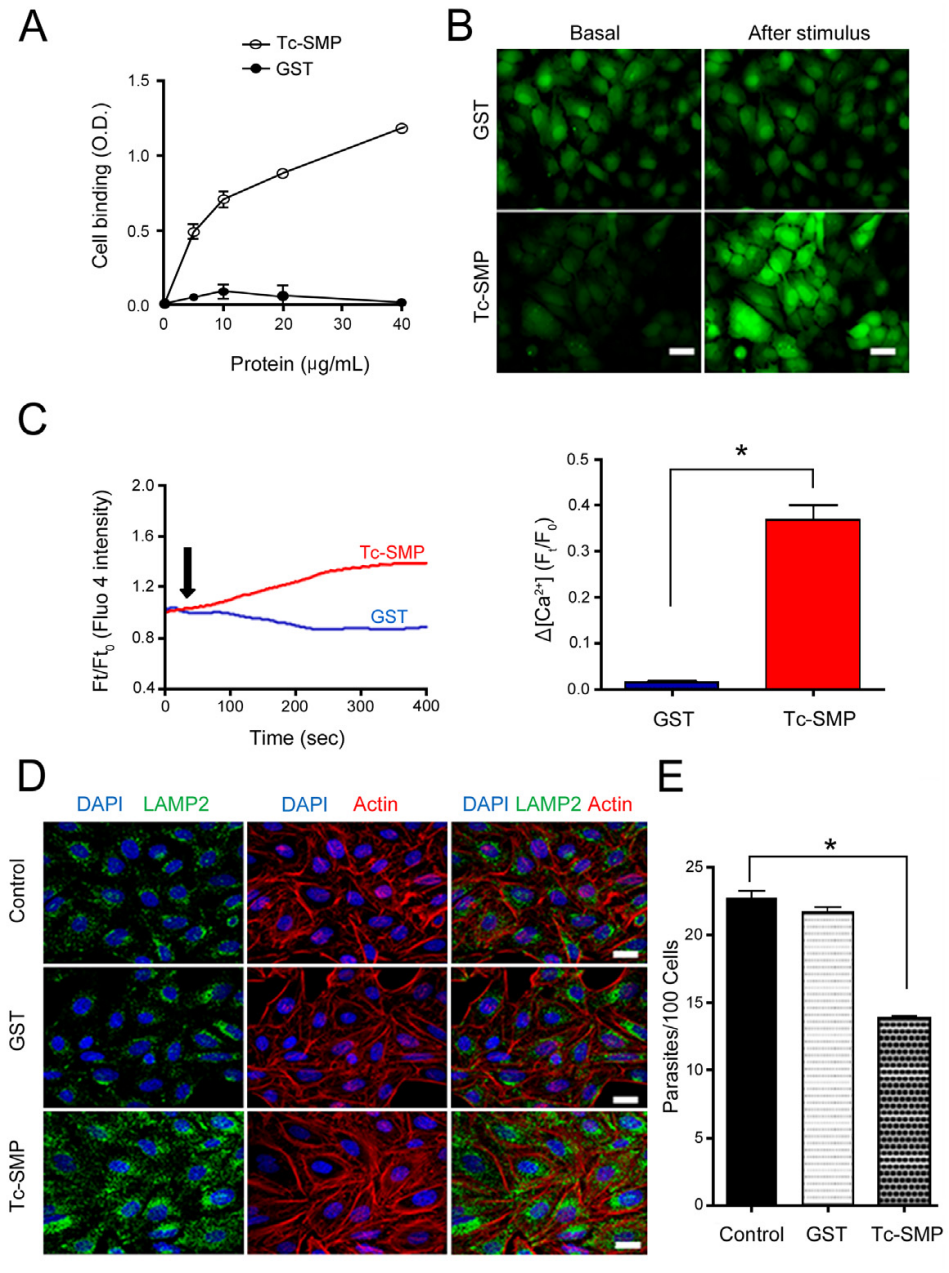
Recombinant protein TcSMP properties in host cell binding, Ca^2+^ signaling, lysosome scattering and inhibition of *T*. *cruzi* metacyclic trypomastigote invasion. A) HeLa cells were incubated with the indicated proteins, at varying concentrations, and binding was evaluated as described in the methods section. Values are the means ± SD of triplicates of one representative assay out of three. B) HeLa cells were grown overnight in DMEM with 10% serum on ibidi multichamber dishes (Hi-Q4, ibidi), and the fluorescence intensity of Fluo-4 was determined as described in the methods section. Images show the basal fluorescence at time zero (left panel) and the maximum fluorescence after stimulation with 40 μg/mL purified TcSMP or GST (right panel). Note the increase in the fluorescence intensity after challenge with TcSMP but not with GST. Bar: 50 μm. C) HeLa cells were grown as in (B) and intracellular calcium was quantified after stimulation (black arrow) with recombinant protein TcSMP or GST. The graph shows the relative concentration of cytoplasmic Ca^2+^ at 400 sec after stimulation, expressed as the maximum peak of total fluorescence minus basal fluorescence at time zero. Thirty cells in three independent experiments were analyzed. Shown on the left side is the difference between the maximum calcium concentration at 400 sec after stimulation with TcSMP and GST (*p = 0.0005). D) HeLa cells were incubated for 30 min with 40 μg/mL of TcSMP or GST, and then processed for confocal fluorescence analysis using anti-LAMP2 antibody and Alexa Fluor 488-conjugated anti-mouse IgG (green), phalloidin-TRITC (red) for actin visualization and DAPI (blue) for DNA, under 63X objective. (Bar: 20 μm). Note the lysosome mobilization to the host cell periphery after treatment with TcSMP. E) HeLa cells were incubated with the indicated recombinant protein at 40 μg/mL. After 30 min, CL strain metacyclic trypomastigotes were added and incubation proceeded for 1 h before fixation and staining with Giemsa. The number of internalized parasites was counted in a total of 250 cells. Values are the means ± SD of three independent assays performed in duplicate. TcSMP, but not GST, significantly inhibited parasite invasion (*p < 0.05).

**Fig 6:**
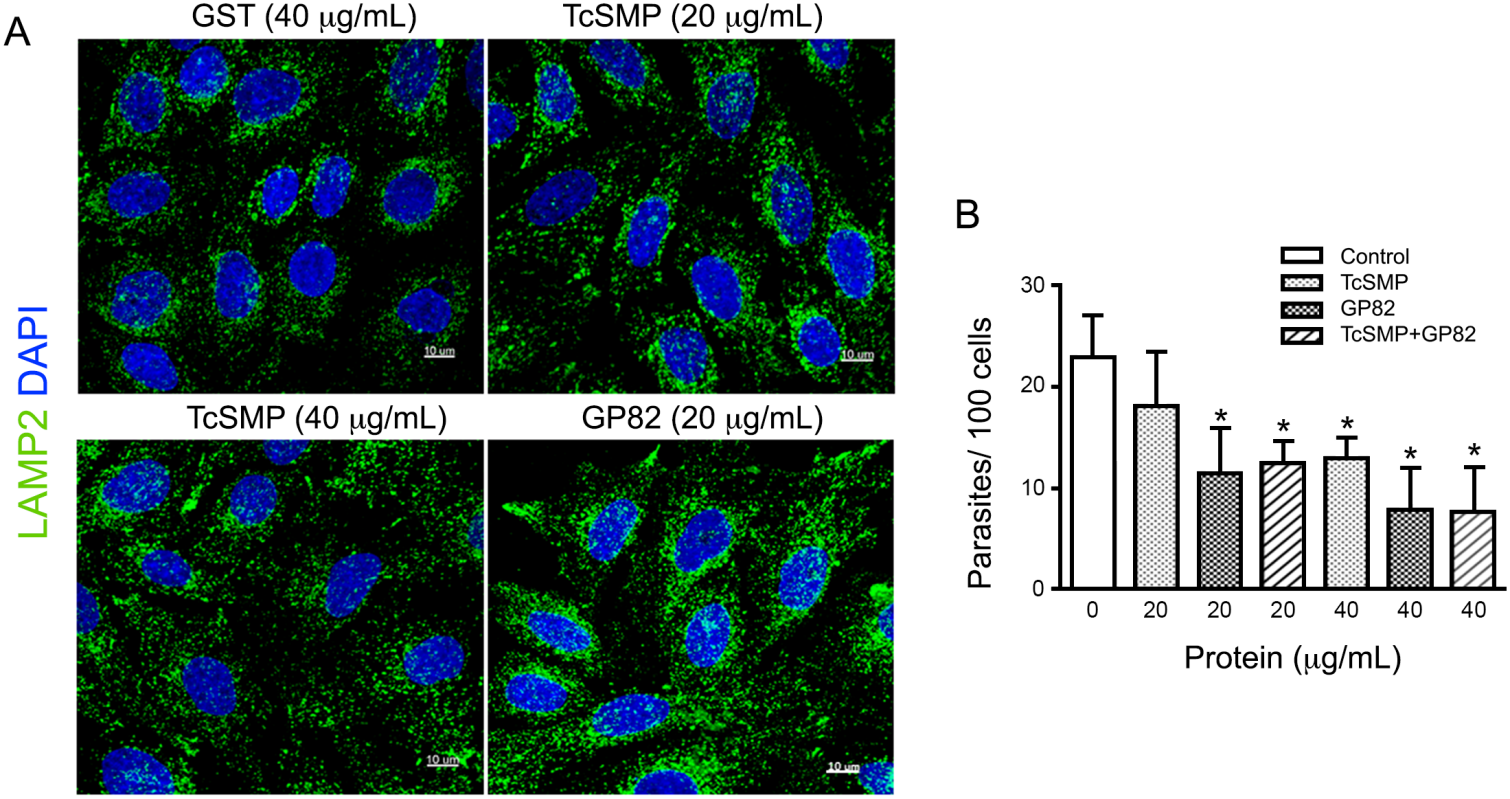
Comparative analysis of lysosome scattering and *T*. *cruzi* cell invasion inhibitory effects of TcSMP and gp82 proteins. A) HeLa cells were incubated for 30 min with TcSMP or gp82, at the indicated concentrations then processed for confocal fluorescence analysis using anti-LAMP2 antibody and Alexa Fluor 488-conjugated anti-mouse IgG (green), and DAPI (blue) for DNA, under 63X objective. (Bar: 10 μm). Note the lysosome mobilization to the host cell periphery after treatment with 40 μg/mL TcSMP. B) HeLa cells were incubated with CL strain metacyclic trypomastigotes in absence or in the presence of TcSMP, gp82 or the combination of the two proteins, at the indicated concentrations. After 1 h incubation, the cells were fixed and stained with Giemsa. The number of internalized parasites was counted in a total of 250 cells. Values are the means ± SD of three independent assays performed in duplicate. Inhibition by TcSMP at 40 μg/mL, by gp82 at 20 μg/mL or 40 μg/mL and by combined proteins, was significant (*p < 0.05).

As the TcSMP protein was detected on the *T*. *cruzi* cell surface (Fig 4), we examined whether it is implicated in parasite-host cell interactions. First, the target cell binding capacity of the recombinant TcSMP protein fused to GST was examined. HeLa cells, immobilized on the bottom of microtiter plates, were incubated with increasing concentrations of recombinant TcSMP or GST, and the bound protein was detected using antibodies directed against TcSMP or GST. Binding of TcSMP to HeLa cells was dose-dependent whereas GST failed to bind (Fig 5A). Next, we determined the ability of the TcSMP protein to trigger host cell Ca^2+^ signaling, which is required for *T*. *cruzi* internalization [44–46]. Recombinant TcSMP or GST, at 40 μg/mL, was added to HeLa cells loaded with the Ca^2+^ indicator fluo-4, and Ca^2+^ signal-inducing activity was monitored by fluorescence microscopy. TcSMP, but not GST, triggered an increase in the fluorescence intensity (Fig 5B). We also evaluated variation of the cytosolic free Ca^2+^ concentration after challenge with 40 μg/mL TcSMP or GST. The recombinant protein TcSMP induced a Ca^2+^ signal that, although moderate, was significant when compared with variation in the fluorescence intensity after stimulation with GST (Fig 5C). As the metacyclic stage surface molecule gp82, which mediates host cell invasion, binds to HeLa cells and induces Ca^2+^ signal [47] and lysosome scattering to the cell periphery followed by exocytosis [48], an event that contributes to parasitophorous vacuole biogenesis [49, 50], we tested whether the TcSMP protein could induce lysosome mobilization. HeLa cells were incubated for 30 min with 40 μg/mL recombinant TcSMP or GST and then processed for immunofluorescence to visualize lysosomes. Scattering of lysosomes from the perinuclear region to the cell periphery was induced by TcSMP protein but not by GST (Fig 5D). The finding that the TcSMP protein triggered Ca^2+^ signaling and lysosome scattering suggested that it could be involved in parasite internalization. To determine the involvement of TcSMP in host cell invasion, assays were performed by incubating CL strain metacyclic forms with HeLa cells for 1 h in the absence or presence of the recombinant TcSMP or GST, at 40 μg/mL. TcSMP protein, but not GST, significantly inhibited parasite invasion (Fig 5E). An additional experiment consisted in comparing the effects of TcSMP and gp82 on lysosome scattering and host cell invasion. HeLa cells were incubated for 30 min with TcSMP or the recombinant gp82, which is also fused to GST, and then processed for immunofluorescence. At 40 μg/mL, TcSMP induced lysosome-scattering comparable to that of gp82 at 20 μg/mL (Fig 6A). The effect of TcSMP at 20 μg/mL was much less pronounced, although it appeared to be higher than that of the GST control (Fig 6A). For cell invasion assays, HeLa cells were incubated for 1 h with CL strain metacyclic forms in the absence or presence of TcSMP or gp82 or in the presence of the two proteins at concentrations of 20 μg/mL and 40 μg/mL. Fig 6B shows that TcSMP significantly inhibited parasite internalization at 40 μg/mL but not at 20 μg/mL, in contrast to gp82, which exhibited an inhibitory effect at 20 μg/mL. The levels of inhibition by the combination of the two proteins were similar to that of gp82 alone (Fig 6B), indicating that the effects of TcSMP and gp82 are not additive.

### Genomic organization of the TcSMP family

**Fig 7:**
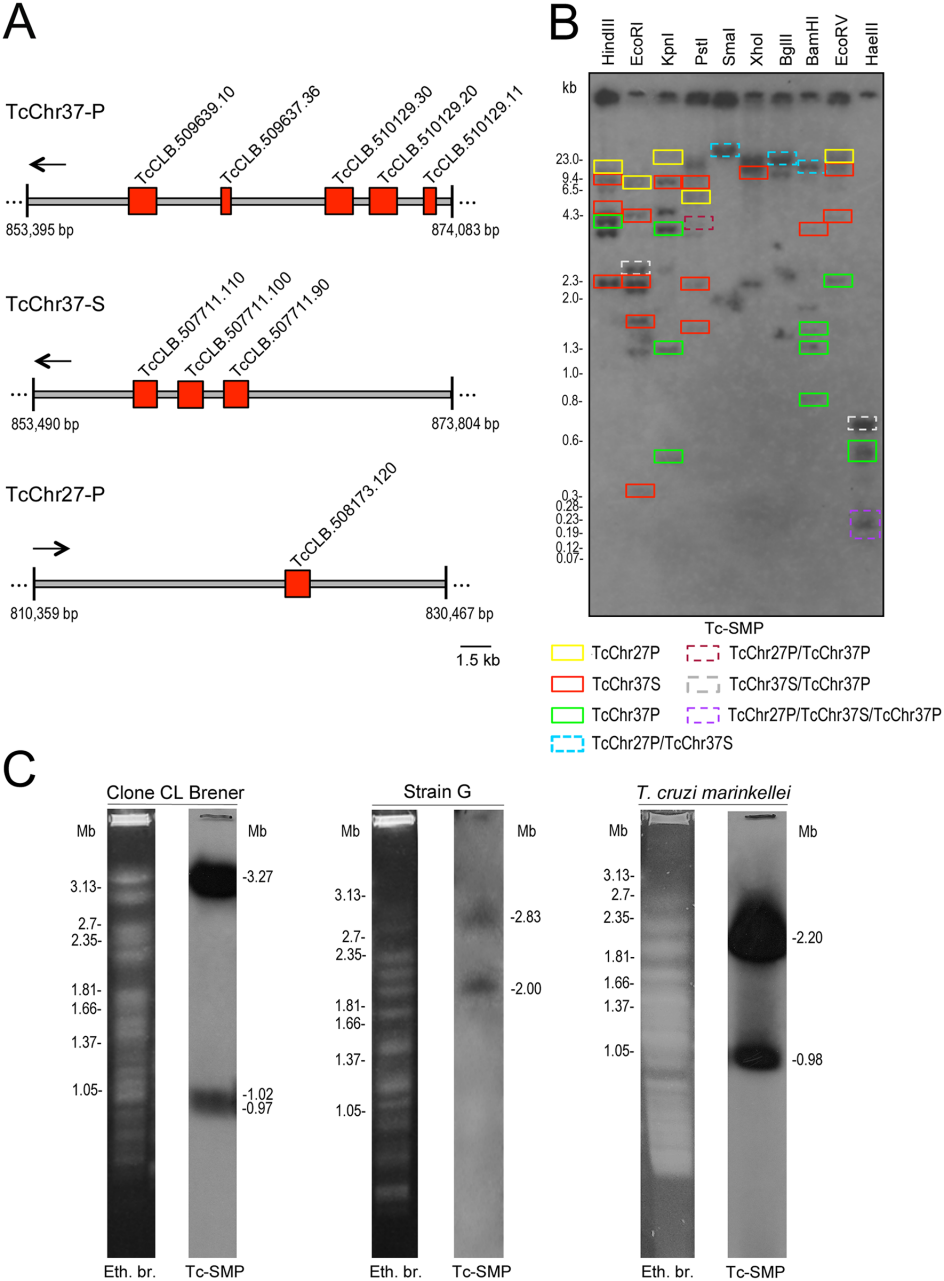
Genomic organization of the TcSMP family. A) Schematic representation of in silico chromosomes TcChr37-P, TcChr37-S and TcChr27-P showing the distribution of TcSMP genes (red boxes). The accession numbers of TcSMP genes in TriTrypDB are indicated above. B) Restriction Southern blot analysis of TcSMP loci. Genomic DNA of CLB was digested with different restriction enzymes, separated on a 0.8% agarose gel and transferred to nylon membrane. Autoradiogram obtained by hybridization with (^32^P) TcSMP probe. The enzymes are indicated above each lane. The colored boxes indicate the restriction fragments predicted in chromosomes TcChr37-P, TcChr37-S, and TcChr27-P. C) Karyotype mapping of TcSMP genes. Chromosomal bands of CLB, strain G and *T*. *cruzi marinkellei* were separated by PFGE, stained with ethidium bromide, transferred onto nylon membranes and hybridized with TcSMP probe. Numbers on the left correspond to the sizes (Mb) of *Hansenula wingei* chromosomes used as markers, and on the right the sizes of chromosomal bands hybridizing with (^32^P) TcSMP probe.

*T*. *cruzi* (CLB) genomic sequences were assembled into 41 chromosome-sized scaffolds designated as TcChr1 to TcChr41 (*T*. *cruzi* in silico chromosomes) [51]. Due to the hybrid nature of clone CL Brener, the chromosome-sized scaffolds were designated S and P to denote the Esmeraldo and non-Esmeraldo haplotypes, respectively [51]. In silico, TcSMP genes were located in chromosomes TcChr37-S, TcChr37-P and TcChr27-P (Fig 7). The homologous chromosomes Tc-Chr37-S and TcChr37P contain a cluster of 3 and 5 TcSMP genes, respectively, while chromosome TcChr27-P contains only one copy. TcSMP genes in the cluster have the same transcriptional orientation (Fig 7A). TcSMP genes located on chromosome TcChr37-P share ≥92% identity with each other or with those in the homologous TcChr37S. Moreover, these sequences exhibit 82–86% identity with TcSMP (TcCLB.508173.120) located on chromosome TcChr27-P.

Due to the in tandem organization of TcSMP genes and high similarity between the sequences, we suggest that TcSMP genes have undergone duplication events followed by mutations that resulted in differences between these genes. Mutation may be one of the mechanisms involved in the generation of sequence diversity and may have contributed to the evolution of these genes in *T*. *cruzi*. Gene duplication and in tandem organization may be important strategies to increase protein production in the absence of transcriptional regulation [52]. TcSMP clusters could have evolved by duplication of a chromosomal region of TcChr37 and dispersion to one chromosomal location in TcChr27.

Restriction-mapping data for the genome sequence in which TcSMP loci are located were utilized to select 10 restriction enzymes that cut inside and/or flanking regions of TcSMP genes. Genomic Southern blot hybridization revealed a relatively simple hybridization profile *c*onsistent with a gene family with few members (Fig 7B). Most computed DNA fragments predicted from *in silico* restriction analysis were superimposed on the images of gels obtained by experimental genomic DNA restriction, as indicated by the colored boxes (Fig 7B). In some cases, the hybridization signals could not be assigned to any fragment predicted by the *in silico* chromosomal digestion analysis. This could be due to the presence of TcSMP genes close to regions of undetermined nucleotides on those chromosomes [51], called N regions, which preclude accurate determination. The novel copies of TcSMP described in this study (KJ682657, KJ682658 and KJ682659) that were not assigned to any chromosome-sized scaffold can also be responsible for not assigned hybridization signals.

The mapping of TcSMP was carried out by chromoblot hybridization with a specific probe that labeled three chromosomal bands of 3.27, 1.02 and 0.97 Mb in CLB (Fig 7C). Our previous data assigned the chromosomes TcChr37-S and TcChr37-P to a chromosomal band of 3.27 Mb, while the chromosome TcChr27-P was assigned to two chromosomal bands of 1.02 and 0.97 Mb [53]. Fig 7C shows the hybridization of the TcSMP probe with two bands in the G strain (2.83 and 2.00 Mb) and in *T*. *cruzi marinkellei* (2.20 and 0.98 Mb), which could correspond to homologous chromosomes with different sizes.

**Fig 8:**
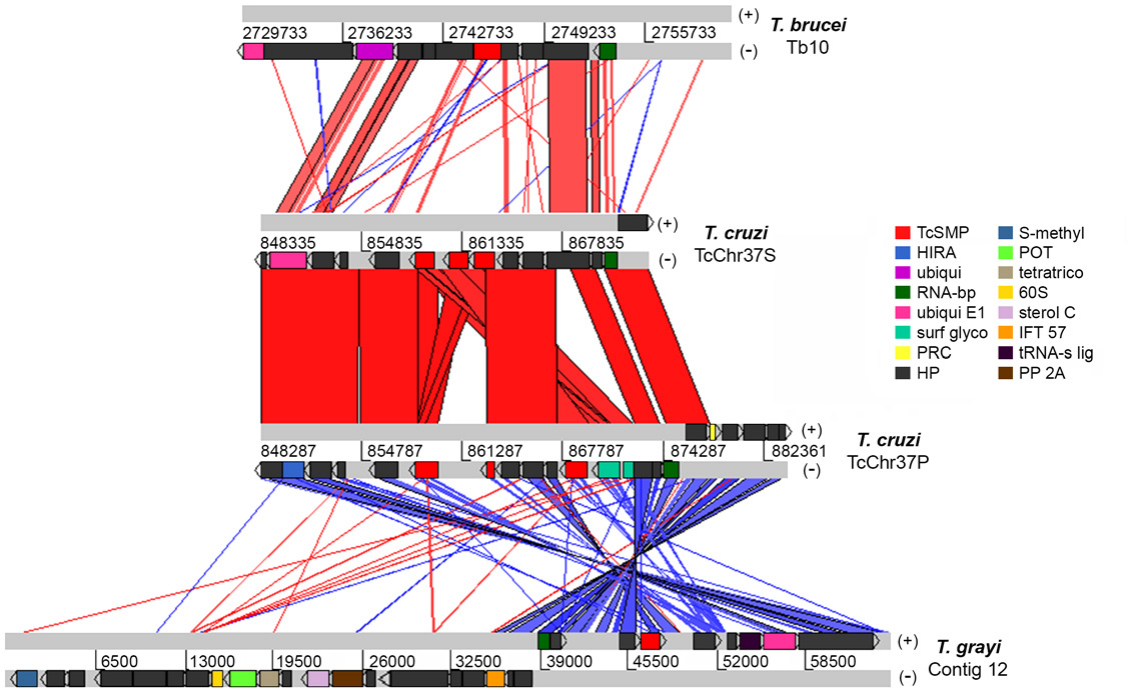
Synteny of TcSMP genes among *T*. *brucei*, T. cruzi and *T*. *grayi*. Genomic regions around the *T*. *cruzi* (CLB) TcSMP paralogs and *T*. *brucei* and *T*. *grayi* orthologs are shown. Analyses were conducted via TBLASTN using the Artemis Comparison Tool (ACT) [37] with an E value of 500. Comparison among TcChr37 (‘‘P” chromosome assigned to the non-Esmeraldo haplotype and ‘‘S” to the Esmeraldo haplotype), *T*. *brucei* chromosome Tb10 and the contig Tgr_12_V1 of *T*. *grayi*. Homologous genes are connected by colored lines. The matches and reverse matches are represented in red and in blue, respectively. Grey blocks represent each chromosome. Chromosome markers are drawn in the sense (+) and antisense (-) strands. The numbers indicate the location on chromosomes: *T*. *brucei* TREU927 (CHR10—position: 2729733–2761348 nt), CLB *T*. *cruzi* (TcChr37-S—positions: 848335–873376 nt and TcChr37-P 848287–882361 nt) and contig of *T*. *grayi* (Tgr_12_V1 –position: 1–64810 nt). The locations of the TcSMP paralogs and *T*. *brucei* and *T*. *grayi* orthologs are depicted in chromosomes by red blocks. Abbreviations: TcSMP (TcSMP—*T*. *cruzi*/ PSSA-2—*T*. *brucei*); HIRA (HIRA-interacting protein 5, putative); ubiqui (ubiquitin-like modifier-activating enzyme ATG7, putative); RNA-bp (RNA-binding protein, putative); ubiqui E1 (ubiquitin activating E1 enzyme, putative); surf glyco (surface glycoprotein, putative); PRC (paraflagellar rod component, putative); HP (hypothetical protein, conserved); S-methyl (S-methyl-5thioribose kinase); POT (proton-dependent oligopeptide transporter, POT family); tetratrico (tetratricopeptide domain 4); 60S (putative 60S ribosomal protein L6); sterol C (sterol C-24 reductase); IFT 57 (predicted: intraflagellar transporter protein 57 homolog); tRNA-s lig (tRNA-splicing ligase RtcB); PP 2A (protein phosphatase 2A regulatory subunit).

In contrast to *T*. *cruzi*, mammal-dwelling African trypanosomes and reptilian trypanosomes appear to have reduced the TcSMP repertoire to a minimum. While *T*. *cruzi* CLB harbors 9 TcSMP genes, *T*. *brucei* and *Trypanosoma grayi* genomes are limited to one ortholog. We compared the corresponding syntenic regions around the TcSMP genes in the genomes of *T*. *cruzi* CLB, *T*. *brucei* and *T*. *grayi*, this last was isolated from African crocodiles (Fig 8). The analysis was performed using a region of approximately 30 kb from chromosome Chr10 of *T*. *brucei*, TcChr37-S and TcChr37-P of *T*. *cruzi* and contig Tgr_12_V1 of *T*. *grayi*. This region exhibits a conserved synteny, the same genes are located in these genomic surroundings in trypanosomes. The conserved genome structure was punctuated by structural divergence, including the insertion/deletion of individual genes and intergenic regions. The bottom of Fig 8 shows the synteny between the chromosome TcChr37P of *T*. *cruzi* and the *T*. *grayi* contig. While conserved synteny relationships between *T*. *cruzi* and *T*. *grayi* can be defined, the exact orientation of *T*. *grayi* genes in relation to *T*. *cruzi* cannot be inferred because the genome of *T*. *grayi* has not yet been assembled. The maintenance of this chromosome region during trypanosome evolution suggests that its genomic organization may be functionally important.

### Phylogenetic tree of TcSMP sequences

**Fig 9:**
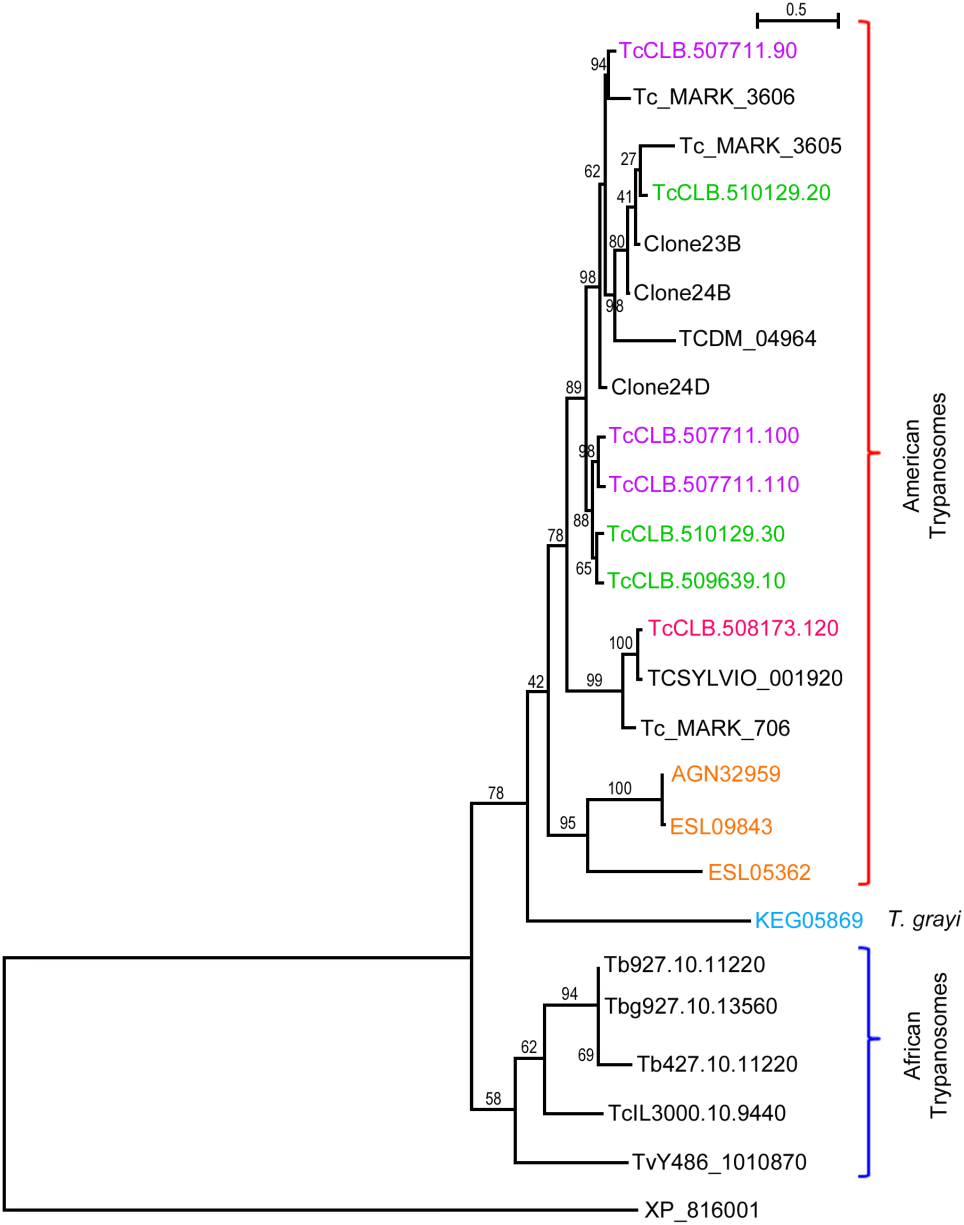
Phylogenetic tree of TcSMP sequences built by the Maximum-Likelihood method. The phylogram was constructed from the alignment of amino acid sequences of TcSMP proteins and their homologs in other trypanosomes. The ML tree was constructed using Phylip. Red and blue lines on the right delimit the American and African trypanosome clusters, respectively. Genes located on chromosome platforms TcChr37-P, TcChr37-S and TcChr27-P are highlighted in green, violet, and pink, respectively. Sequences were identified by their entries in TriTrypDB, except those from *T*. *rangeli* (AGN32959, ESL09843 and ESL05362) and *T*. *grayi* (KEG05859), which were identified by their GenBank accession numbers. TriTrypDB entries are prefixed by species and strain followed by the number of the sequence. *T*. *cruzi*: TcCLB., clone CL Brener; TCDM_, clone Dm28c; TCSylvio_, clone Sylvio X10/1; *T*. *cruzi marinkellei*: Tc_MARK_; *T*. *brucei brucei*: Tb927., strain TREU; Tb427., strain Lister; *T*. *vivax*: TvY486_; *T*. *congolense*: TcIL3000. Clones 23B, 24B and 24D (GenBank KJ682657, KJ682658 and KJ682659) were isolated in this work by PCR amplification using TcSMP specific primers. A trans-sialidase from *T*. *cruzi* CLB (XP_816001) was used as the outgroup.

A phylogenetic tree calculated from the alignment of TcSMP sequences, excluding truncated sequences, is shown in Fig 9. TcSMP proteins appear to have followed different evolutionary pathways, with two main branches for the clustering of American and African trypanosomes. Within the American trypanosomes, sequences located on the same *T*. *cruzi* chromosomes tended to cluster on the same branch: Tc.CLB.510129.30 and TcCLB.509639.10 belonging to TcChr37-P (highlighted in green) and TcCLB.507711.100 and TcCLB.507711.110 belonging to TcChr37-S (highlighted in purple), excluding two peptides that did not cluster with the others (TcCLB.510129.20 belonging to TcChr37-P and TcCLB.507711.90 belonging to TcChr37-S). It is worth noting that the TcSMP sequences misannotated as TS in the CLB (TcCLB.508173.120), clone Sylvio (TCSYLVIO_001920) and *T*. *cruzi marinkellei* (Tc_MARK_706) genomes tended to group on the same branch, showing that these sequences may exhibit some differences compared to other TcSMP peptides. *T*. *vivax*, *T*. *congolense* and *T*. *brucei brucei*-*T*. *brucei gambiense* and *T*. *brucei* proteins are clustered into three separate branches. *T*. *brucei* species comprises three morphologically indistinguishable subspecies *T*. *b*. *gambiense*, *T*. *b*. *rhodesiense* and *T*. *b*. *brucei* [54]. Studies based on SSU and 5.8S ribosomal sequences had indicated the location of *T*. *vivax* branch marginal to the *T*. *brucei*, *T*. *b*. *gambiense* and *T*. *congolense* branch [55].

*T*. *grayi* and *T*. *ralphi* were in sister subclades. *T*. *grayi*, a crocodilian trypanosome, clustered apart from American and mammal-dwelling African trypanosomes. Interestingly, *T*. *grayi* was closer to *T*. *cruzi*, *T*. *c*. *marinkellei* and *T*. *rangeli* than to African trypanosomes (*T*. *brucei gambiense*, *T*. *congolense* and *T*. *vivax*). The subclade *T*. *grayi* comprised trypanosomes from African crocodilids and tsetse flies [56, 57], while the subclade *T*. *ralphi* comprised trypanosomes from South American alligatorids represented by *T*. *ralphi* sp. from crocodilian caimans of Brazilian river basins [58].”

## Discussion

We demonstrated that TcSMP is a novel gene family that is conserved among different *T*. *cruzi* lineages and with orthologs in other *Trypanosoma* species, including *T*. *grayi*, a trypanosome of reptiles. Genome annotation update is an important step to validate the accuracy and relevancy of genetic information and may provide new information about genomic structure and organization as well as gene function. In the *T*. *cruzi* databases (TcruziDB and GenBank) TcSMP genes were annotated as surface glycoproteins or procyclic form surface phosphoproteins and a few were annotated as trans-sialidases. An extensive body of literature exists regarding the structure and function of highly expressed *T*. *cruzi* surface proteins (mucin, TS, cruzipain, amastin) [10, 12–14, 59] however, little information is available for the less abundant proteins (MASP; TcTASV, DGF1, SAP) [19–24, 60–62]. Recently large-scale experimental approaches comprising multistep protein separation strategies and proteomic analysis allowed the identification of a number of low abundance proteins.

TcSMP proteins were first identified by proteomic analysis of enriched membrane-enriched fractions of *T*. *cruzi* isolated by phase partitioning with Triton X-114 [27]. Several observations led us to conclude that TcSMP is a membrane-spanning protein located at the cellular surface and is also released to the extracellular milieu. First, the deduced amino acid sequence of TcSMP showed the key elements typical of surface proteins in trypanosomes, namely the presence of an N-terminal signal peptide or a signal anchor and a C-terminal hydrophobic sequence predicted to be a TM domain (Fig 1 and S5 Fig). Second, immunofluorescence of live parasites with anti-TcSMP antibodies clearly labeled the surface of all *T*. *cruzi* developmental forms (Fig 4). Co-localized pixels between TcSMP-GFP and anti-BIP in confocal images obtained by immunofluorescence of parasites expressing TcSMP-GFP suggests that TcSMP goes to the ER and then it is addressed to the cell surface membrane (S2B Fig). Moreover, anti-TcSMP antibodies also reacted with PSSA-2, an ortholog expressed at the cell surface of *T*. *brucei* procyclic forms (S6 Fig). Third, TcSMP peptides, previously found in a membrane-enriched fraction, were also identified by proteomic analysis in membrane vesicles as well as in a soluble form in the secretome of epimastigote and metacyclic forms (Table 2). Taken together, our results are compatible with the assumption that TcSMP are membrane-spanning proteins located at the outer surface and can be released into the extracellular milieu. TcSMP proteins are also located intracellularly in all developmental forms, likely associated with membrane-bound structures (Fig 4).

TcSMP_S and TcSMP_L are α-helical transmembrane proteins that appear to differ from one another by the mechanism with which they are translocated to the plasma membrane (Fig 1 and S5 Fig). TcSMP_S and TcSMP_L have 2–3 TM spanning domains therefore, they could be classified as polytopic proteins inserted into the plasma membrane through two TM domains (S5 Fig). TcSMP_L has three TM domains, the first is a canonical amino-terminal signal peptide that should target the protein to the ER lumen following cleavage by the signal peptidase. TcSMP_S contains two TM domains, the first domain is also found after the initiator methionine and it has been predicted to be a non-cleavable signal anchor. Prediction of membrane protein topology suggested that TcSMP_L are proteins with the N- and C-terminus outside (e.g., TcCLB.510129.20) or inside (e.g., TCD_04964, Tc_MARK_306, Tc_MARK_706) and TcSMP_S are proteins with N- and C-terminal inside (S5 Fig). Of the seven TcSMP proteins identified in *T*. *cruzi* (clone CLB), four have a similar topology to TCCLB510129.20, i.e., the larger hydrophilic domain, located in the middle of the protein, is predicted to be located intracellularly. The remaining three TcSMP proteins of CLB have the opposite topology, which means that the larger hydrophilic domain is predicted to be located extracellularly. This topology has also been predicted in other *T*. *cruzi* isolates (clones DM28c, Sylvio X/10), *T*. *cruzi marinkellei* and *T*. *brucei*. Anti-SMP antibodies were generated against 101 amino acid sequences of the N-terminal domain, comprising the second TM domain (22 aa) and a segment of 79 aa that is exposed to the outer surface in TcSMP_S and several TcSMP_L proteins (TCD_04964, Tc_MARK_3605, Tc_MARK_706). The reaction to live parasites by the anti-TcSMP antibodies confirmed that TcSMP is exposed on the outer surface. Anti-TcSMP antibodies also reacted with live *T*. *brucei* parasites confirming that PSSA-2 is attached to the outer surface of the plasma membrane by a stable TM anchor [25, 26]. Although our findings favor the model in which the larger hydrophilic middle domain is predicted to be located extracellularly, we cannot rule out the presence of both TcSMP protein topologies in the parasite. In African trypanosomes and *T*. *rangeli*, the orthologs have two TM domains, and the first functions as a signal anchor (Fig 2). TcSMP orthologs in *T*. *grayi*, a reptilian trypanosome, have a single TM domain at the carboxy-terminal domain.

TcSMP proteins are expressed in all *T*. *cruzi* developmental stages. Translation of TcSMP mRNAs resulted in a single protein with an apparent molecular mass of 40 kDa, indicating that TcSMP proteins have the same molecular mass. The apparent molecular mass of native TcSMP (40 kDa) on SDS-PAGE is relatively close to that predicted after the processing of TcSMP (41.3 to 43.7 kDa). If we assume that the TcSMP_L are processed from the signal peptide and the TcSMP_S from the first methionine and signal anchor, they should have fairly similar molecular masses (41.3 to 43.7 kDa). The observed discrepancies between the predicted and the observed SDS-PAGE mobility could be explained in part by the cleavage of a short sequence at the amino-terminal region. Another possibility could be the targeting of the nascent polypeptide to the endoplasmic reticulum through the anchor signal where it would be recognized and cleaved by a signal peptide peptidase. The experimental evidence indicates that polytopic membrane proteins can be cleaved internally by a signal peptidase that recognizes non-canonical sequences [63] which could explain the smaller size of the native protein. Finally, it remains unclear whether an individual trypanosome expresses a single TcSMP gene or co-expresses different members of the TcSMP family.

Several pieces of evidence have indicated that the TcSMP protein is implicated in host cell invasion. Similar to the surface molecule gp82, which mediates the internalization of metacyclic trypomastigotes, TcSMP bound to target cells and induced Ca^2+^ signaling and lysosome mobilization (Fig 5), events that are required for parasitophorous vacuole biogenesis [48, 49]. In addition, the recombinant TcSMP protein was capable of inhibiting metacyclic trypomastigote entry into host cells (Fig 5). The effects of the TcSMP protein on target cell lysosome scattering and parasite invasion were found to be of lower magnitude compared to gp82, suggesting that TcSMP may play an auxiliary role in parasite invasion, as was previously ascribed to the SAP protein. Like TcSMP, the SAP protein bound to host cells and triggered Ca^2+^ signaling and lysosome exocytosis, and its recombinant form exhibited the property of inhibiting metacyclic trypomastigote invasion [19, 24]. We envisage the possibility that the productive interaction of *T*. *cruzi* with host cells that effectively results in internalization may depend on diverse adhesion molecules. In the case of metacyclic forms, the signaling induced by TcSMP and SAP may add to that triggered by the major surface molecule gp82, further increasing the host cell responses required for infection.

TcSMP genes are densely clustered within a ∼16-kb region of the genome, with one cluster composed of five genes on chromosome TcChr37-P and another composed of 3 genes on TcChr37-S (Fig 7). Each of the TcSMP genes in a cluster is in the same transcription orientation. Adjacent TcSMP genes located within the same chromosome share 91–98% identity, and similar values were obtained for TcSMP sequences located on homologous chromosomes TcChr37-P and TcChr37-S. This family could have originated by tandem gene duplication of an ancestral gene and sequence homogenization. The solitary TcSMP copy was found in the chromosome TcChr27-P, and it could have arisen by gene duplication followed by translocation to this chromosome. The degree of sequence similarity and synteny found among TcSMP and its orthologs indicates that the TcSMP and surrounding regions existed before the branching of the evolutionary tree that resulted in different species of the *Trypanosoma* genus. The maintenance of this chromosome region during trypanosome evolution suggests that its genomic organization may be functionally important.

The role of TcSMP in the invasion of mammalian cells by trypomastigotes was demonstrated in several experiments. However, the reason why all developmental forms express TcSMP proteins remains unclear. TcSMP may have another role in non-invasive *T*. *cruzi* forms e.g., to work as a sensor for parasite interaction with the environment of the invertebrate host similarly to the function suggested for its ortholog PSSA-2 in *T*. *brucei* [26]. Although we found orthologous TcSMP genes in several other trypanosome species, there is no evidence of expression of this protein in these protozoans. A detailed, functional characterization should therefore be carried out to explain the role of this protein in extracellular and non-invasive forms.

TcSMP can be added to the repertoire of proteins expressed at the cell surface and also secreted by *T*. *cruzi*. Although it is a minor component of the cell coat when compared to GPI-anchored molecules such as TS, mucins and GIPLs, it has a role in the invasion of mammalian cells by metacyclic trypomastigotes. TcSMP could also transmit signals to host cells. Recently, proteins secreted by *T*. *cruzi* have been implicated in arrhythmias in an *ex-vivo* model [64]. It has been suggested that pro-arrhythmogenic proteins secreted or released by *T*. *cruzi* could act as enhancers causing the cardiac conduction system to cross an arrhythmic threshold in chagasic patients. Thus, characterization of a novel gene family encoding proteins that can be found at the cell coat or secreted by *T*. *cruzi* can provide new understanding of the interaction of this parasite with its host cells.

## Supporting Information

S1 Fig Alignment of TcSMP sequences of clone CL Brener (CLB) by the MegAlign program (DNASTAR). Sequences TcCLB510129.20, TcCLB510129.30, TcCLB509639.10, TcCLB507711.90, extended TcCLB507711.100, extended TcCLB507711.110 and TcCLB507711.120 are from TriTrypDB. Clones 23B, 24B and 24D (GenBank KJ682657, KJ682658 and KJ682659) were isolated in this work by PCR amplification using TcSMP specific primers indicated in the figure. Primers used in the PCR reaction are indicated with asterisks below the alignment (Forward: P1F and P2F; Reverse: P3R and P4R). Identical residues are highlighted in black; 80% identity in dark gray and 60% identity in light gray.
(ZIP)

S2 Fig Additional controls to check antibody specificity and cellular location of TcSMP protein. A) Western assays using anti-TcSMP or anti-MVK antibodies against the purified TcSMP-GST and MVK (mevalonate kinase) recombinant proteins. Molecular mass markers in kilodaltons (kDa) are indicated on the left and reactive protein molecular masses on the right. B) Cellular distribution of TcSMP protein in cells transfected with the construct pTREX-TcSMP-GFP. Parasites expressing TcSMP-GFP (green) were incubated with anti-TcSMP or anti-BIP (reticulum marker) antibodies followed by incubation with Alexa Fluor 488-labeled anti-mouse immunoglobulin (red). Confocal images obtained from each fluorescence channel were overlapped, and co-localized pixels are shown in panel CP. Parasite DNA was stained with DAPI (blue). Differential interference contrast (DIC) images are shown on the left. Scale bar, 2 μm.
(TIF)

S3 Fig Extended TcCLB.507711.100 and TcCLB.507711.110 nucleotides and amino acids sequences. Nucleotide and translated sequences of TcCLB.507711.100 and TcCLB.507711.110 genes including 132- and 102-bp-extensions at the 5’ terminus (in bold) that were missed in the automatic annotation. Note that 44 and 34 amino acids (in bold) were added to N-termini of the TcCLB.507711.100 and TcCLB.507711.110 proteins, respectively.
(TIF)

S4 Fig Identification of Kozak consensus sequence in TcSMP_L. The nucleotide sequences of the 5’ end of the TcSMP_L genes of clone CL Brener were aligned by the MegAlign program (DNASTAR). The accession numbers of the TcSMP sequences in TriTrypDB are TcCLB510129.20, TcCLB510129.30, TcCLB509639.10, extended TcCLB.507711.100 and extended TcCLB.507711.110. Black, dark gray and light gray highlighted regions share 100%, 80% and 60% identity, respectively. Red and violet boxes indicate the translation start codon (methionine codon) and consensus Kozak sequence, respectively.
(TIF)

S5 Fig Prediction of transmembrane domains in TcSMP proteins and PSSA-2 using the TMHMM program. The hydrophobicity plot was predicted following modeling by the "Hidden Markov Model" (HMM) with the TMHMM program. Red peaks indicate the transmembrane domains. The predicted topology is indicated by blue and pink colors, representing intracellular and extracellular domain, respectively. TcSMP_L proteins have 3 hydrophobic regions, the first one corresponds to the signal peptide predicted by SignalP 3.0, here represented by TcCLB510129.20 (CLB) and TCDM_04964 (Dm28c). TcSMP_S proteins have 2 hydrophobic regions, the first one corresponds to a putative signal anchor predicted by SignalP 3.0, here represented by TcCLB507711.90 (CLB); Tb927.10.11220 (PSSA-2 from *T*. *brucei*) and ESL05362 (*T*. *rangeli*). Clone 23B (KJ682657) were isolated from the genomic DNA of CLB by PCR with TcSMP specific primers. The Frag-I fragment was expressed as a recombinant protein in *E*. *coli*.
(TIF)

S6 Fig Histogram showing the fluorescence intensity pattern of *T*. *cruzi* tissue culture trypomastigotes (TCT) and *T*. *brucei* procyclic forms stained with anti-SMP serum in non-permeabilized live (upper) or permeabilized fixed (lower) parasites. The red and blue lines indicate parasites incubated with anti-SMP and pre-immune mouse (*T*. *cruzi*) and rabbit (*T*. *brucei*) sera, respectively. Control experiments of *T*. *brucei* flow cytometry experiment were performed with parasites incubated without antibodies (black line) or only with the antibody conjugated to the fluorophore (green line).
(TIF)
