# Supplementary figures and images for: Molecular Characterization of a Novel Family of Trypanosoma cruzi Surface Membrane Proteins (TcSMP) Involved in Mammalian Host Cell Invasion
Source: PLoS Negl Trop Dis. 2015 Nov 13;9(11):e0004216. doi: 10.1371/journal.pntd.0004216 (PMC4643927; doi:10.1371/journal.pntd.0004216)

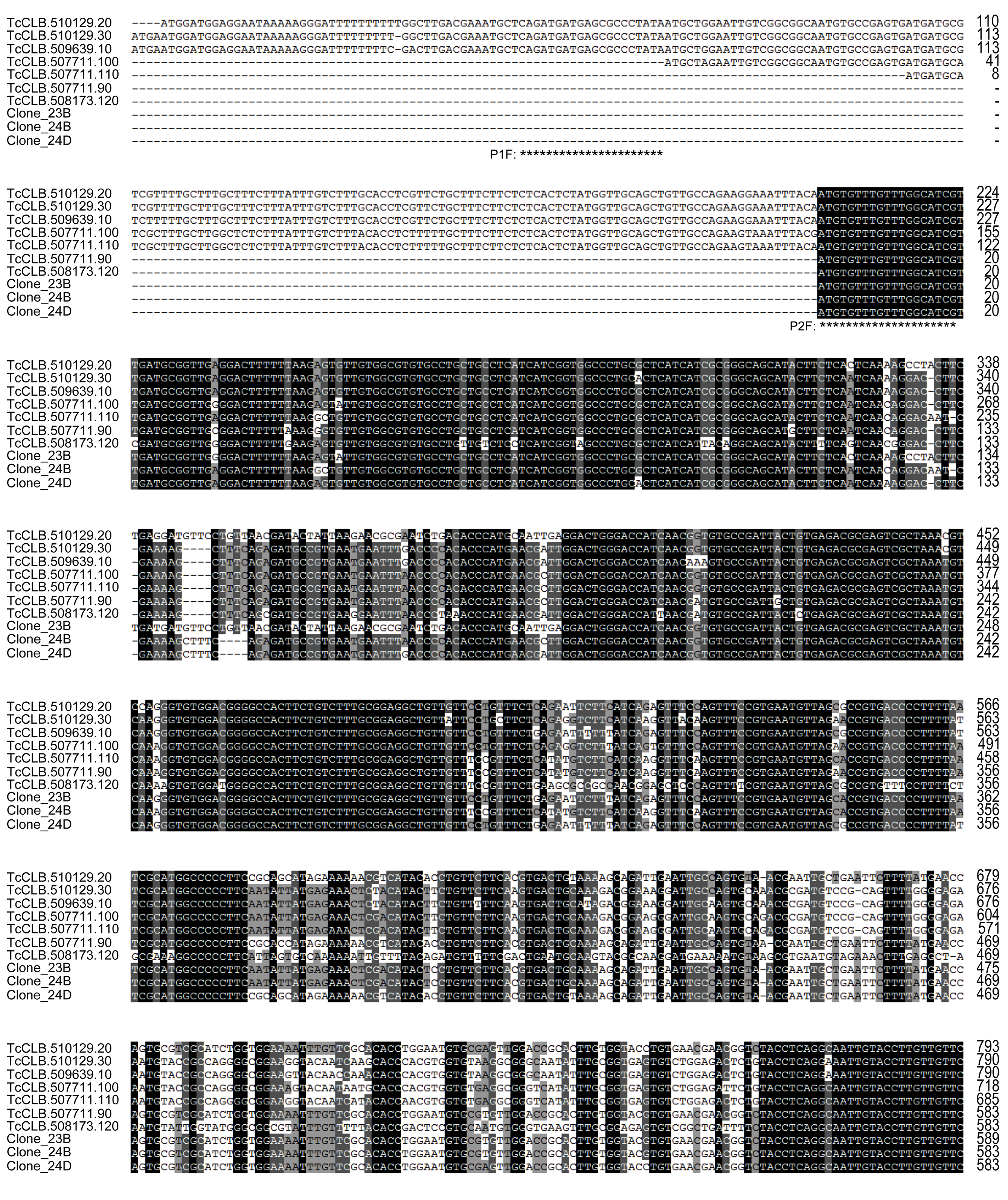

Supplement: S1 Fig — Sequences TcCLB510129.20, TcCLB510129.30, TcCLB509639.10, TcCLB507711.90, extended TcCLB507711.100, extended TcCLB507711.110 and TcCLB507711.120 are from TriTrypDB. Clones 23B, 24B and 24D (GenBank KJ682657, KJ682658 and KJ682659) were isolated in this work by PCR amplification using TcSMP specific primers indicated in the figure. Primers used in the PCR reaction are indicated with asterisks below the alignment (Forward: P1F and P2F; Reverse: P3R and P4R). Identical residues are highlighted in black; 80% identity in dark gray and 60% identity in light gray. (ZIP) [file pntd.0004216.s001.zip › FigS1_page1.tif]

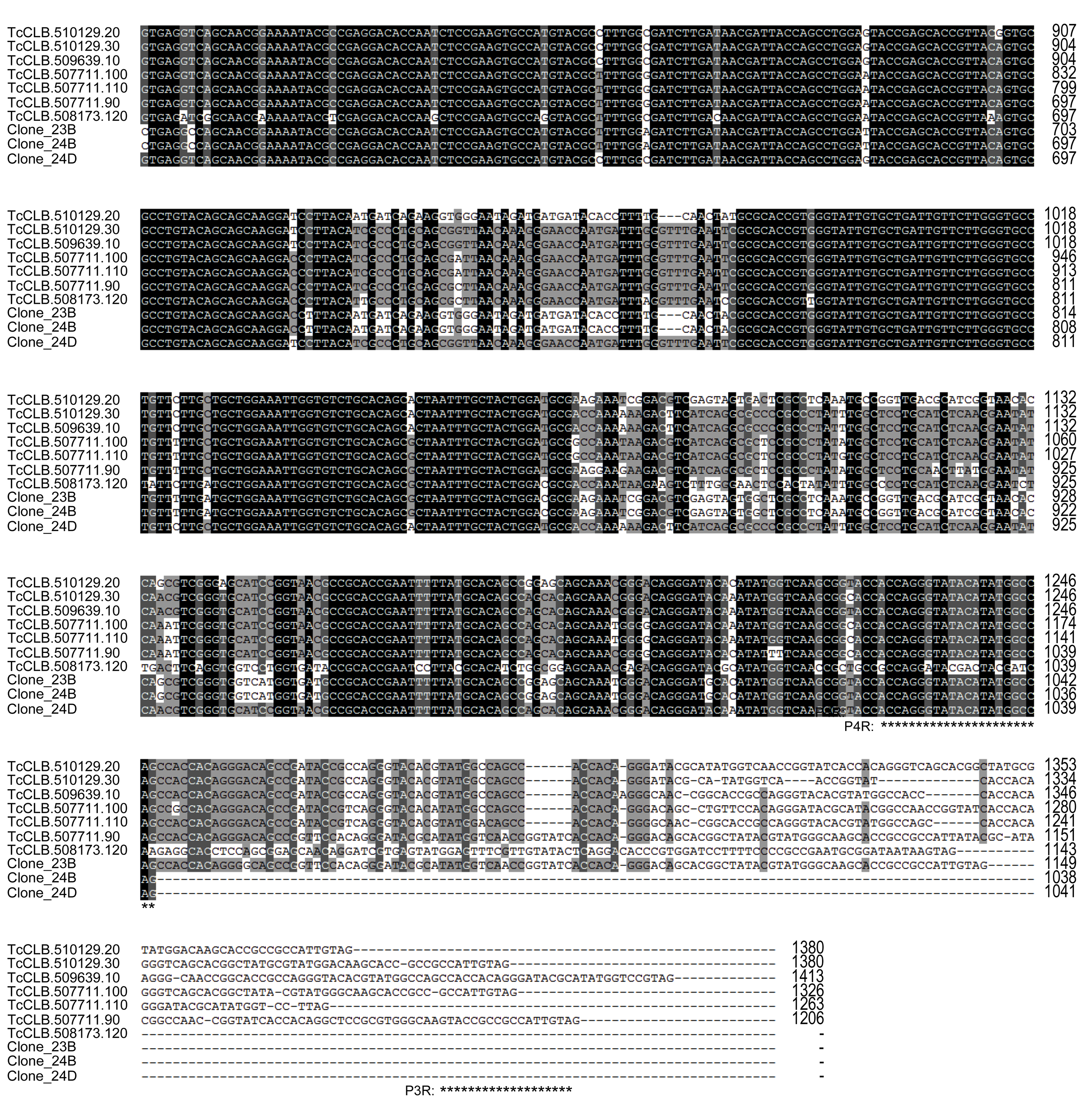

Supplement: S1 Fig — Sequences TcCLB510129.20, TcCLB510129.30, TcCLB509639.10, TcCLB507711.90, extended TcCLB507711.100, extended TcCLB507711.110 and TcCLB507711.120 are from TriTrypDB. Clones 23B, 24B and 24D (GenBank KJ682657, KJ682658 and KJ682659) were isolated in this work by PCR amplification using TcSMP specific primers indicated in the figure. Primers used in the PCR reaction are indicated with asterisks below the alignment (Forward: P1F and P2F; Reverse: P3R and P4R). Identical residues are highlighted in black; 80% identity in dark gray and 60% identity in light gray. (ZIP) [file pntd.0004216.s001.zip › FigS1_page2.tif]

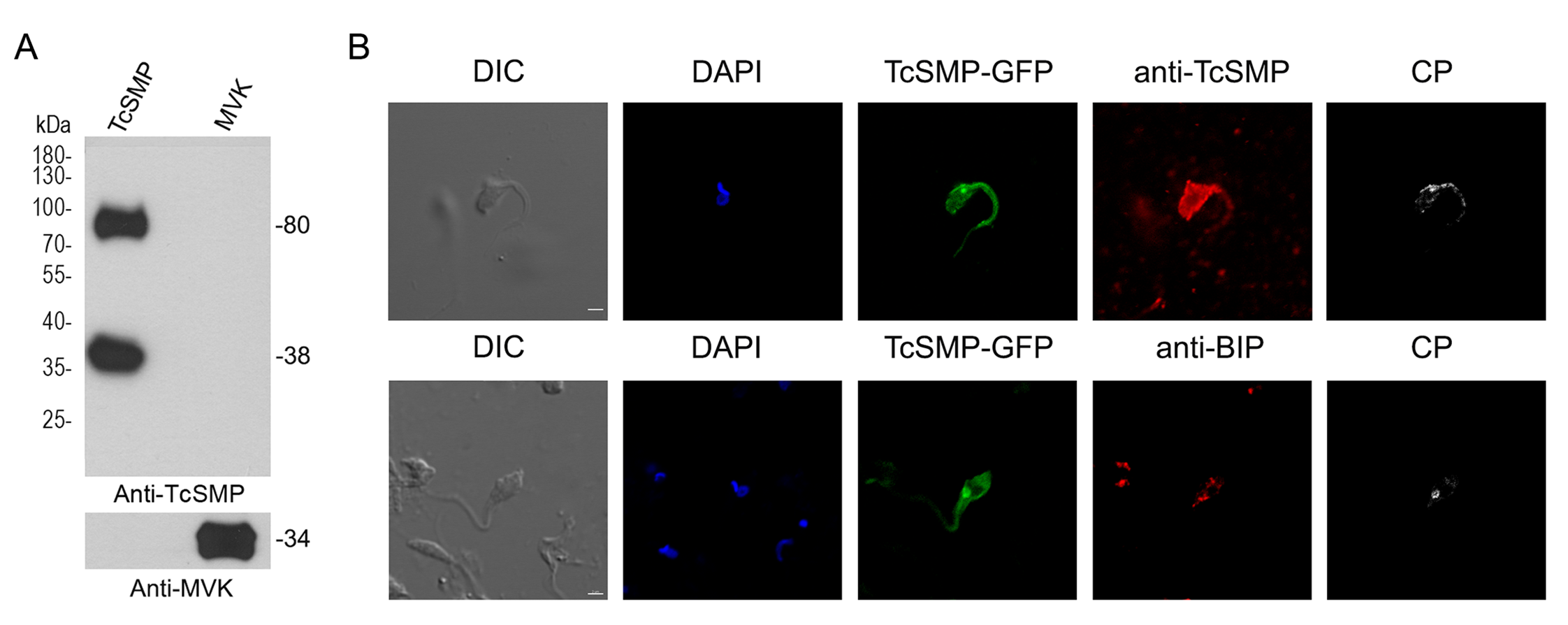

Supplement: S2 Fig — A) Western assays using anti-TcSMP or anti-MVK antibodies against the purified TcSMP-GST and MVK (mevalonate kinase) recombinant proteins. Molecular mass markers in kilodaltons (kDa) are indicated on the left and reactive protein molecular masses on the right. B) Cellular distribution of TcSMP protein in cells transfected with the construct pTREX-TcSMP-GFP. Parasites expressing TcSMP-GFP (green) were incubated with anti-TcSMP or anti-BIP (reticulum marker) antibodies followed by incubation with Alexa Fluor 488-labeled anti-mouse immunoglobulin (red). Confocal images obtained from each fluorescence channel were overlapped, and co-localized pixels are shown in panel CP. Parasite DNA was stained with DAPI (blue). Differential interference contrast (DIC) images are shown on the left. Scale bar, 2 μm. (TIF) [file pntd.0004216.s002.tif]

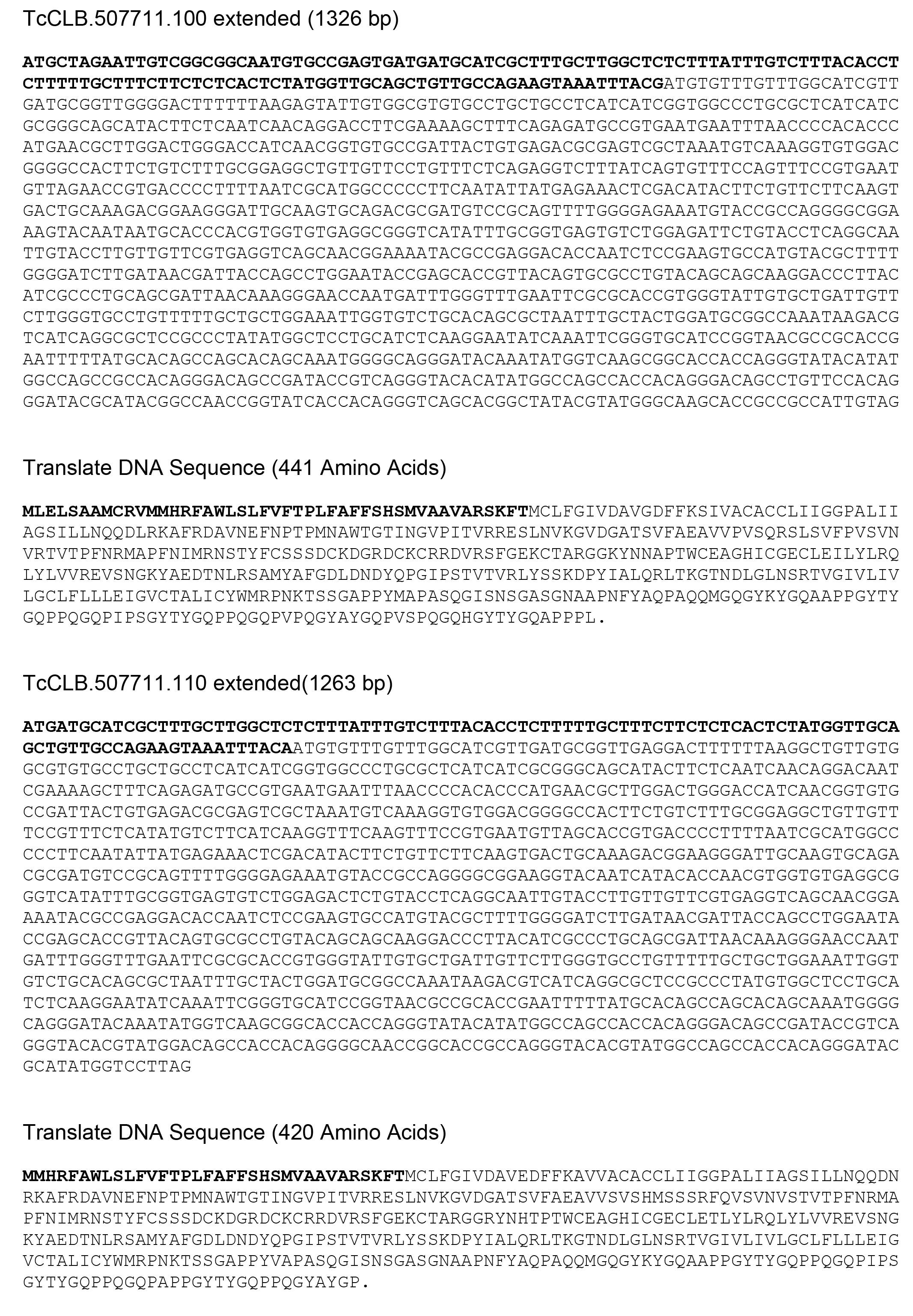

Supplement: S3 Fig — Nucleotide and translated sequences of TcCLB.507711.100 and TcCLB.507711.110 genes including 132- and 102-bp-extensions at the 5’ terminus (in bold) that were missed in the automatic annotation. Note that 44 and 34 amino acids (in bold) were added to N-termini of the TcCLB.507711.100 and TcCLB.507711.110 proteins, respectively. (TIF) [file pntd.0004216.s003.tif]

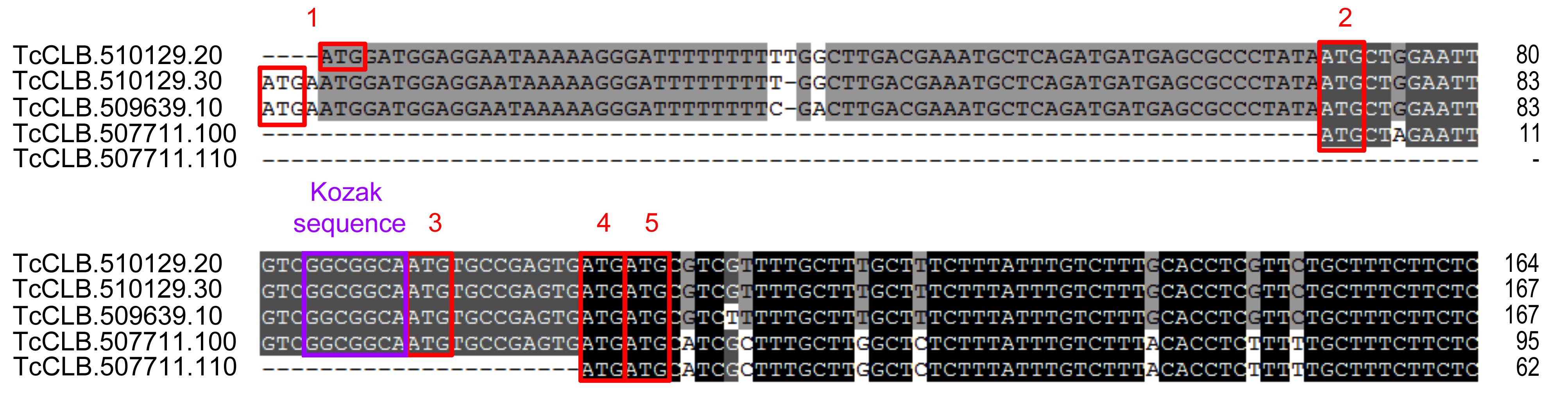

Supplement: S4 Fig — The nucleotide sequences of the 5’ end of the TcSMP_L genes of clone CL Brener were aligned by the MegAlign program (DNASTAR). The accession numbers of the TcSMP sequences in TriTrypDB are TcCLB510129.20, TcCLB510129.30, TcCLB509639.10, extended TcCLB.507711.100 and extended TcCLB.507711.110. Black, dark gray and light gray highlighted regions share 100%, 80% and 60% identity, respectively. Red and violet boxes indicate the translation start codon (methionine codon) and consensus Kozak sequence, respectively. (TIF) [file pntd.0004216.s004.tif]

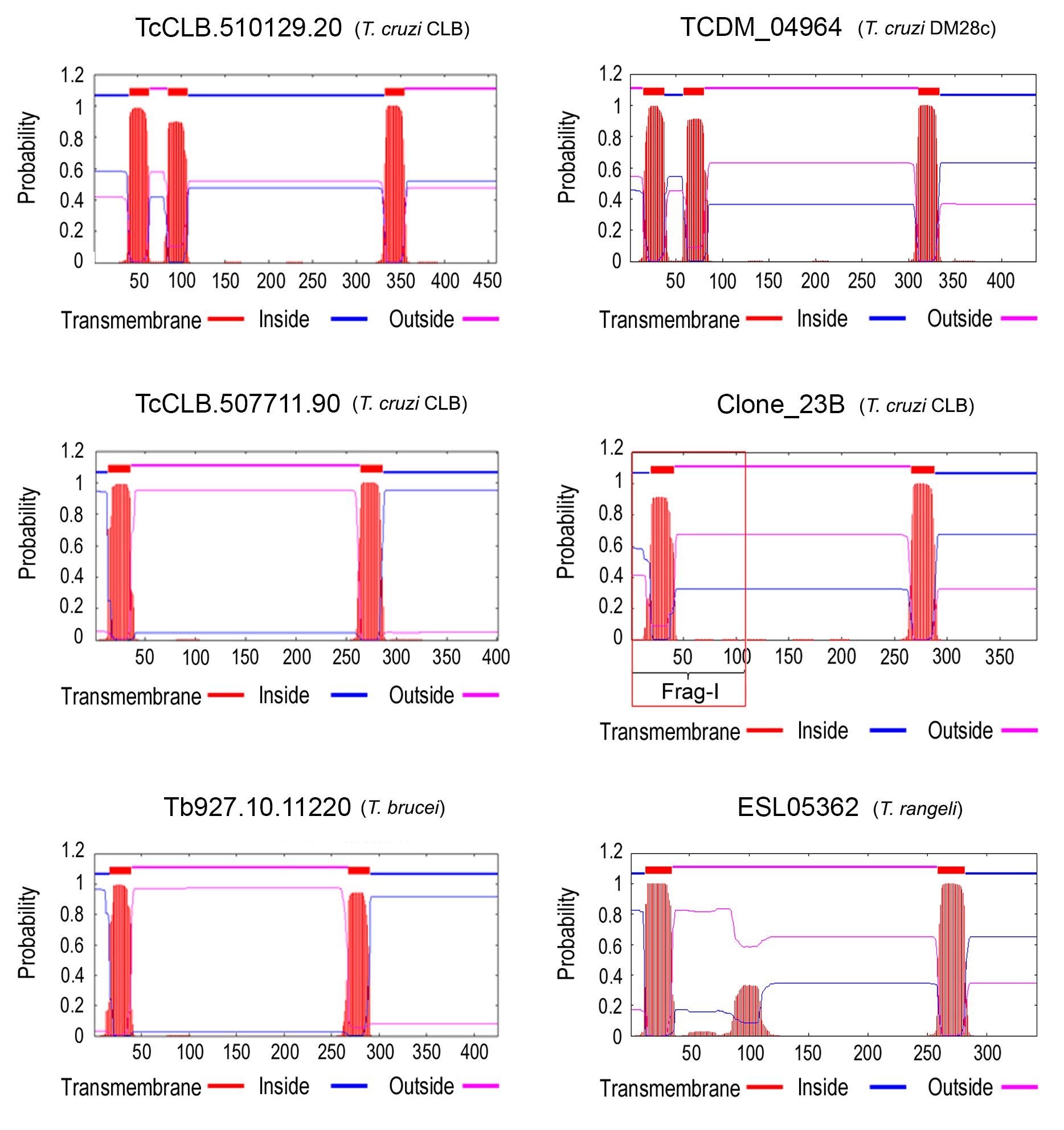

Supplement: S5 Fig — The hydrophobicity plot was predicted following modeling by the "Hidden Markov Model" (HMM) with the TMHMM program. Red peaks indicate the transmembrane domains. The predicted topology is indicated by blue and pink colors, representing intracellular and extracellular domain, respectively. TcSMP_L proteins have 3 hydrophobic regions, the first one corresponds to the signal peptide predicted by SignalP 3.0, here represented by TcCLB510129.20 (CLB) and TCDM_04964 (Dm28c). TcSMP_S proteins have 2 hydrophobic regions, the first one corresponds to a putative signal anchor predicted by SignalP 3.0, here represented by TcCLB507711.90 (CLB); Tb927.10.11220 (PSSA-2 from T. brucei) and ESL05362 (T. rangeli). Clone 23B (KJ682657) were isolated from the genomic DNA of CLB by PCR with TcSMP specific primers. The Frag-I fragment was expressed as a recombinant protein in E. coli. (TIF) [file pntd.0004216.s005.tif]

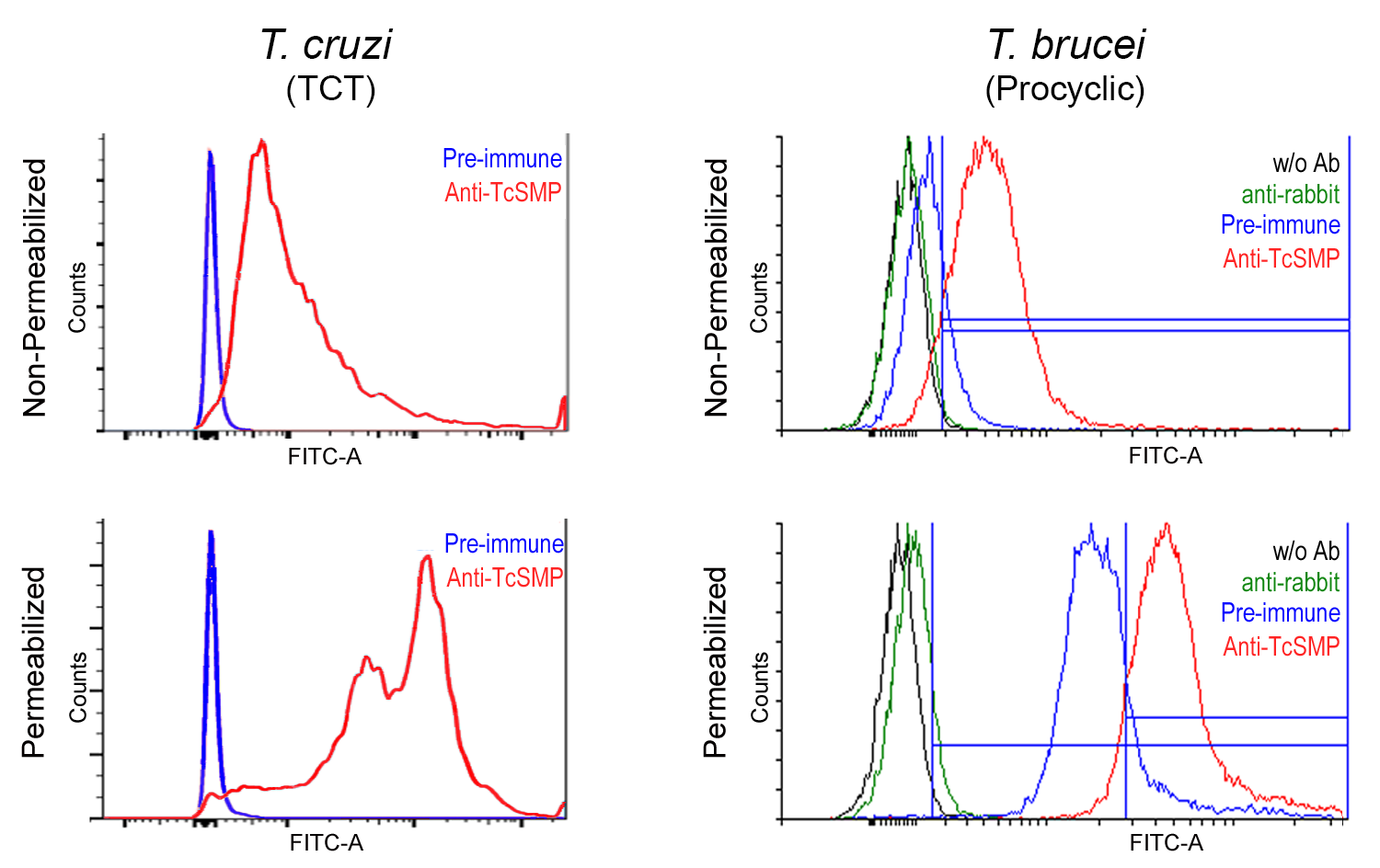

Supplement: S6 Fig — The red and blue lines indicate parasites incubated with anti-SMP and pre-immune mouse (T. cruzi) and rabbit (T. brucei) sera, respectively. Control experiments of T. brucei flow cytometry experiment were performed with parasites incubated without antibodies (black line) or only with the antibody conjugated to the fluorophore (green line). (TIF) [file pntd.0004216.s006.tif]
